# Morphological and molecular characterisation of *Scutellonema* species from yam (*Dioscorea* spp.) and a key to the species of the genus

**DOI:** 10.1163/15685411-00003084

**Published:** 2017-07-11

**Authors:** Yao A. Kolombia, Gerrit Karssen, Nicole Viaene, P. Lava Kumar, Lisa Joos, Danny L. Coyne, Wim Bert

**Affiliations:** 1 Nematology Research Unit, Department of Biology, Ghent University, K.L. Ledeganckstraat 35, B-9000 Ghent, Belgium; 2 International Institute of Tropical Agriculture (IITA), PMB 5320, Oyo Road, Ibadan, Nigeria; 3 National Plant Protection Organization, 6706 EA Wageningen, The Netherlands; 4 Flanders Research Institute for Agriculture, Fisheries and Food (ILVO), B-9820 Merelbeke, Belgium; 5 IITA, Kasarani, P.O. Box 30772-00100, Nairobi, Kenya

**Keywords:** *COI*, D2-D3, diagnostics, Ghana, identification, key, Nigeria, phylogeny, *Scutellonema cavenessi*, *Scutellonema clathricaudatum*, *Scutellonema paralabiatum*, species delimitation, West Africa

## Abstract

The yam nematode, *Scutellonema bradys*, is a major threat to yam (*Dioscorea* spp.) production across yam-growing regions. In West Africa, this species cohabits with many morphologically similar congeners and, consequently, its accurate diagnosis is essential for control and for monitoring its movement. In the present study, 46 *Scutellonema* populations collected from yam rhizosphere and yam tubers in different agro-ecological zones in Ghana and Nigeria were characterised by their morphological features and by sequencing of the D2-D3 region of the 28S rDNA gene and the mitochondrial *COI* genes. Molecular phylogeny, molecular species delimitation and morphology revealed *S. bradys, S. cavenessi, S. clathricaudatum* and three undescribed species from yam rhizosphere. Only *S. bradys* was identified from yam tuber tissue, however. For barcoding and identifying *Scutellonema* spp., the most suitable marker used was the *COI* gene. Additionally, 99 new *Scutellonema* sequences were generated using populations obtained also from banana, carrot, maize and tomato, including the first for *S. paralabiatum* and *S. clathricaudatum*, enabling the development of a dichotomous key for identification of *Scutellonema* spp. The implications of these results are discussed.

Yam (*Dioscorea* spp.) is an important staple crop cultivated for its edible tubers in West Africa (Asiedu & Sartie, 2010). The plant-parasitic nematode *Scutellonema bradys* (Steiner & LeHew, 1933) Andrássy, 1958, or ‘the yam nematode’, is a migratory endoparasite that causes dry rot disease of yam tubers, creating a persistent decline of tuber quality and even total loss during storage (Bridge *et al.*, 2005). Feeding by the nematode results in necrotic lesions beneath the outer skin. These lesions become yellow and gradually brown to black with progression of the disease. The outer skin may be intact, disguising the damage below, or it may become flaky or develop cracks, which serve to facilitate secondary infection by fungi and bacteria causing wet rot (Ekundayo & Naqvi, 1972; Demeaux *et al.*, 1982).

In the root and soil environment, *S. bradys* cohabits with many closely related and morphologically similar species (Sher, 1964; Bridge *et al.*, 1995; Coyne *et al.*, 2012), which creates difficulties in diagnostics (Baujard & Martiny, 1995). Accurate species identification is necessary for determining pest management options and for monitoring and surveillance activities to establish distribution, movement and quarantine measures. When screening for resistance in yam, correct and accurate identification of the target pest is also essential.

*Scutellonema* spp. are associated with roots of a wide range of crops (Sher, 1964; Van den Berg & Heyns, 1973; Knight, 2001; Agudelo & Harshman, 2011; Coyne *et al.*, 2016). The genus *Scutellonema* was proposed by Andrássy (1958) and included all *Rotylenchus* members with large phasmids (scutella) located either opposite each other or nearly so and at the level of the anus or cloacal aperture. In a comprehensive review, Sher (1964) listed 11 species, a list later expanded to 45 valid *Scutellonema* species (Siddiqi, 2000). Just three new species have since been reported (Saha *et al.*, 2000; Giribabu & Saha, 2002). Species-level identification has traditionally relied upon detailed morphological analysis, a lengthy and labour intensive task that requires considerable expertise (Coomans, 2000) given the morphological conservatism within a genus (Powers *et al.*, 2011). *Scutellonema* spp. identification is based on the analysis of morphometrics and morphological characters, such as lip region morphology, lip region shape, number of lip region annuli, number of striations on the basal lip annulus, position of the hemizonid, secretory-excretory (S-E) pore and scutella, size of the scutella, structure of the female reproductive system, presence of ‘vaginal glands’ (conspicuous cuticular thickenings towards ends of vulva) and epiptygmata, and areolation at scutella level (Sher, 1964; Smit, 1971; Van den Berg & Heyns, 1973; Germani *et al.*, 1985a; Baujard *et al.*, 1990; Krall, 1990). However, given the lack of tangible morphological characters to distinguish important *Scutellonema* species, *viz*., *S. bradys, S. cavenessi* Sher, 1964 and *S. clathricaudatum* Whitehead, 1959a, Baujard & Martiny (1995) grouped these three species into the “*S. bradys* complex”.

DNA barcoding-based methods have proved invaluable for delineating species lacking contrasting morphological features. The DNA regions coding for ribosomal genetic markers (D2-D3 of 28S rRNA-, 18S-, and ITS- rRNA) have been commonly used to identify *Scutellonema* spp. (Chen *et al*, 2006; Subbotin *et al.*, 2007; Van den Berg *et al.*, 2013, 2017; Tzortzakakis *et al.*, 2016). The mitochondrial Cytochrome c Oxidase I gene (mt*COI*) (Hebert *et al.*, 2003), which is commonly used for barcoding, has also been explored for a limited number of nematode species (Palomares-Rius *et al.*, 2014), including *Scutellonema* spp. (Van den Berg *et al.*, 2013, 2017).

The aims of this study were: *i*) to conduct species level characterisation of *Scutellonema* populations collected from yam tubers and yam rhizosphere in the main yam growing areas in Nigeria and Ghana, using morphological, morphometric and molecular data (D2-D3 expansion segments of 28S rDNA gene and Cytochrome c oxidase subunit 1 (*COI*); *ii*) to determine the phylogenetic interrelations to delimit species; and *iii*) to develop a morphological key for species of *Scutellonema*.

## Materials and methods

### Nematode Samples

Nematode populations used in this study were isolated from yam rhizosphere and yam tubers taken from farmers’ fields and experimental plots in different agro-ecological zones in Ghana and Nigeria during surveys conducted between 2012 and 2015 (Table 1). Nematode populations from soil, roots and tubers were isolated using the White-head and Hemming tray technique (see Hooper *et al.*, 2005). Soil samples of 100 ml were used for nematode extraction. Yam roots retrieved from each soil sample were carefully washed, chopped into small pieces (0.5-2.0 cm) and processed separately from the soil. For tubers, three subsamples of 5 g were used for the extraction from yam peel (Coyne *et al.*, 2006; Baimey *et al.*, 2009). Nematode populations isolated from various substrates were collected on 28 *μ*m sieves, washed, and divided into two parts for preservation for further analysis: one part was heat-killed and fixed in 4% formalin; the other was fixed directly in DESS solution (Yoder *et al.*, 2006). Altogether, 120 rhizosphere and 84 tuber isolates were collected for species identification studies.

**Table 1:** Source of Scutellonema materials used for characterising the genus.

Country	State/District	Locality	Latitude (°W)	Longitude (°N)	Altitude (m asl)	Host	Sample code	Isolate	Species	GenBank accession no.	
										D2-D3 of 28S rRNA	COI
Ghana	East Gonja	Adamupe	8.49292	−0.51155	176	*Dioscorea rotundata*	4GR28-1-1	K212	*Scutellonema* sp. 1		KY639362
		8.49292	−0.51155	176	*D. rotundata*	4GS28-1-2	K213	*Scutellonema* sp. 1	KY639319		
			8.49302	0.51146	180	*D. rotundata*	4GS27-1	K303	*Scutellonema* sp. 1		KY639364
			8.49302	0.51146	180	*D. rotundata*	4GS27-1-d5	K304	*Scutellonema* sp. 1		KY639365
			8.49302	0.51146	180	*D. rotundata*	4GS27-1-n2	K325	*Scutellonema* sp. 1		KY639366
			8.49302	0.51146	180	*D. rotundata*	4GS27-1-n3	K326	*Scutellonema* sp. 1	KY639320	
			8.49302	0.51146	180	*D. rotundata*	4GS27-1-d-n2	K328	*Scutellonema* sp. 1	KY639321	KY639367
	Kintampo	Kintampo	8.04879	−1.69498	326	*D. alata*	L17-3	K86	*S. clathricaudatum*	KY639301	KY639343
	Kintampo North	Bablioduo Konkomba	8.03699	−1.86572	273	*D. rotundata*	4GS13-1-1	K211	*S. bradys*	KY639282	KY639329
			8.0352	−1.86789	265	*D. rotundata*	4GR12-1-1	K218	*Scutellonema* sp. 1		KY639363
		Kintampo Sogliboi	8.14838	−1.84069	209	*D. rotundata*	4GS15-1-2	K227	*Scutellonema* sp. D		KY639371
	Tolon	Akukayele	9.39046	−1.00179	197	*D. rotundata*	4GR22-2-1	K221	*S. clathricaudatum*		KY639352
			9.39046	−1.00179	197	*D. rotundata*	4GS17-1-a1	K290	*Scutellonema* sp. D		KY639383
			9.39046	−1.00179	197	*D. rotundata*	4GS17-1-n1	K321	*Scutellonema* sp. D	KY639323	
		Baturoyili	9.46677	−1.14103	150	*D. alata*	L28-1	K87	*S. clathricaudatum*	KY639314	KY639355
			9.46677	−1.14103	150	*D. alata*	L28-2	K88	*S. clathricaudatum*	KY639315	KY639356
		Kpalsogu	8.14838	−1.84069	171	*D. rotundata*	4GS18-16-3	K236	*S. bradys*		KY639377
		Kunguri	9.53501	−1.13915	149	*D. alata*	L29-1	K89	*S. clathricaudatum*	KY639310	KY639350
		Nyanpala	9.40463	−0.92124	150	*D. rotundata*	L31-1	K91	*Scutellonema* sp. 1	KY639318	
		Wala	9.63993	−1.24929	124	*D. rotundata*	4GS22-1-2	K204	*S. clathricaudatum*	KY639311	KY639379
			9.63993	−1.24929	124	*D. rotundata*	4GS22-1-7	K208	*S. clathricaudatum*	KY639312	KY639351
			9.63993	−1.24929	124	*D. rotundata*	4GR22-2-2	K222	*S. clathricaudatum*	KY639313	KY639382
			9.63993	−1.24929	124	*D. rotundata*	4GR22-2-4	K224	*S. clathricaudatum*		KY639353
	Unknown	Unknown	–	–	–	*D. rotundata* T	*2T1-4*	K53	*S. bradys*	KY639281	KY639328
Nigeria	Abia	Umuagu	5.60529	7.44844	83	*D. alata*	4NS5-3-1	K132	*S. bradys*		KY639334
			5.61234	7.44739	90	*D. dumetorum*	2NS16-5-3	K99	*S. cavenessi*	KY639294	
			5.61234	7.44739	90	*D. dumetorum*	2NS16-5-4	K100	*S. cavenessi*	KY639295	KY639338
			5.61234	7.44739	90	*D. dumetorum*	2NS16-5-6	K101	*S. cavenessi*	KY639296	
Nigeria	Abia		5.61234	7.44739	90	*D. rotundata*	2NS16-1-2	K93	*S. cavenessi*		KY639340
			5.61234	7.44739	90	*D. rotundata*	2NS16-1-3	K94	*S. cavenessi*	KY639299	KY639341
		Umudiawa	5.6076	7.43423	90	*D. alata*	2NS15-9-1	K70	*S. cavenessi*	KY639292	KY639337
			5.6076	7.43423	90	*D. alata*	2NS15-9-2	K71	*S. cavenessi*	KY639293	
		Umudike	5.48212	7.53057	108	*D. alata*	4NR6-4-1	K135	*Hoplolaimus* sp.	KY639326	KY639374
			5.48559	7.53173	150	*D. rotundata*	2NS13-1-3	K38	*S. cavenessi*	KY639297	KY639339
			5.48559	7.53173	150	*D. rotundata*	2NS13-1-2	K39	*S. cavenessi*	KY639298	
	Abuja	Gwagwalada	8.92592	7.09447	198	*D. rotundata*	2NS32-1-1	K80	*Scutellonema* sp. D	KY639325	KY639373
			8.79809	7.08173	244	*D. rotundata* T	*3M20-1-3*	K68	*S. bradys*	KY639288	
		Kilankwa	8.88516	7.09258	232	*D. rotundata*	4NS11-1-1	K122	*S. clathricaudatum*	KY639309	KY639348
		Old Chukuku	8.8778	7.13536	145	*D. rotundata*	2NS30-1-2	K79	*Scutellonema* sp. D	KY639324	KY639372
	Benue	Otobi	7.11317	8.10366	137	*D. rotundata*	2NS23-9-2	K41	*Scutellonema* sp. 2	KY639322	KY639368
			7.11317	8.10366	137	*D. rotundata*	2NS23-13-1	K103	*Scutellonema* sp. 2		KY639369
			7.11317	8.10366	137	*D. rotundata*	2NS23-13-2	K104	*Scutellonema* sp. 2		KY639370
		7.11798	8.1023	136	*D. rotundata*	4NS9-1-1	K128	*S.*	*clathricaudatum*		KY639349
Edo	Aviele	7.00446	6.27694	103	*D. rotundata*	2NS7-1-2	K17	*S.*	*clathricaudatum*		KY639354
	Uromi	6.71174	6.32965	398	*D. rotundata* T	*3M10-1-1*	K29	*S.*	*bradys*	KY639284	KY639331
Ekiti	Oye	7.79962	5.3304	548	*D. rotundata* T	*3M2-5-4*	K59	*S.*	*bradys*	KY639286	KY639333
		7.79962	5.33039	548	*D. rotundata* T	*3M2-5-5*	K61	*S.*	*bradys*	KY639287	
Imo	Owerri	5.5915	7.2876	158	*D. rotundata* T	*YOS39*	K188	*S.*	*bradys*	KY639289	KY639335
		5.5915	7.2876	158	*D. rotundata* T	*YOS40*	K189	*S.*	*bradys*	KY639290	KY639336
		5.5915	7.2876	158	*D. rotundata* T	*YOS49*	K198	*S.*	*bradys*	KY639291	KY639378
Nasarawa	Jidna	9.06625	7.62225	440	*D. rotundata*	2NS29-1-1	K42	*S.*	*clathricaudatum*	KY639304	
		9.06625	7.62225	440	*D. rotundata*	2NS29-13-2	K45	*S.*	*clathricaudatum*	KY639305	KY639346
		9.06625	7.62225	440	*D. rotundata*	2NS29-19-1	K47	*S.*	*clathricaudatum*	KY639306	KY639347
		9.06625	7.62225	440	*D. rotundata*	2NS29-5-4	K50	*S.*	*clathricaudatum*	KY639307	
		9.06625	7.62225	440	*D. rotundata*	2NS29-7-5	K78	*S.*	*clathricaudatum*	KY639308	
	Rimuka	8.49365	8.51599	175	*D. rotundata* T	*3M17-8-1*	K32	*S.*	*bradys*	KY639285	KY639332
	Oyo	Bodija	7.43506	3.9143	221	*D. cayenensis* T *4M11-3*	K297	*S.*	*bradys*	KY639283	KY639330
Nigeria	Oyo	Ibadan	7.49402	3.8973	205	*D. rotundata* T	*YT1*	–	*S. bradys*		
		Tafo	8.61064	3.46563	441	*D. alata*	2NS35-9-1	K74	*S. clathricaudatum*		KY639344
			8.61064	3.46563	441	*D. alata*	2NS35-9-2	K75	*S. clathricaudatum*	KY639303	KY639345
			8.61064	3.46563	441	*D. cayenensis*	2NS35-5-1	K84	*S. clathricaudatum*		KY639302
Ethiopia	West Arsi zone	Hajjee area	–	–	–	*Zea mays*	–	AD3	*S. paralabiatum*		KY639358
	West Arsi zone	Wondo Genet	–	–	–	*Z. mays*	–	AD1	*S. paralabiatum*		KY639357
Rwanda	−	Zone 3	–	–	–	*Allium cepa*	–	AL31	*S. cavenessi*		KY639381
	–		–	–	–	*Musa* sp.	–	AL7	*S. brachyurus*		KY639327
	–		–	–	–	*Musa* sp.	–	AL8	*S. paralabiatum*		KY639361
	–		–	–	–	*Musa* sp.	–	AL34	*S. paralabiatum*		KY639317
	–	Zone 4	–	–	–	*Allium cepa*	–	AL4	*S. paralabiatum*		KY639360
	–	Zone 8	–	–	–	*A. cepa*	–	AL1	*S. cavenessi*		KY639342
	–	Zone 8	–	–	–	*A. cepa*	–	AL2	*S. paralabiatum*		KY639359
	–	Zone 8	–	–	–	*A. cepa*	–	AL30	*S. paralabiatum*		KY639316 KY639380
	–	Zone 8	–	–	–	*Daucus carota*	–	AL26	*S. cavenessi*		KY639300
	–	Zone 8	–	–	–	*Solanum lycopersicum*	–	AL37	*Tylenchorhynchus* sp.		KY639375
	–	Zone 8	–	–	–	*S. lycopersicum*	–	AL38	*Tylenchorhynchus* sp.		KY639376

Host names followed by the letter T are samples from yam tuber; otherwise, samples each soil sample is from a composite of the rhizosphere of four plants.

### Morphological Characterisation

Nematode specimens fixed in formalin were processed to anhydrous glycerin following the glycerin-ethanol method (Seinhorst, 1959) as modified by De Grisse (1969). Permanent slides were prepared and used to record morphometrics and morphological features (Sher, 1964; Germani *et al.*, 1985a; Krall, 1990; Van den Berg *et al.*, 2013) using an Olympus BX51 DIC microscope equipped with a Nikon digital camera. Additional morphological and morphometric data were recorded from temporary slides made from DESS fixed specimens, prior to DNA extraction. In addition, paratypes and other populations of the genus *Scutellonema*, available in the nematode collections in Ghent University Museum – Zoology Collections, Belgium (UGent), and in the Wageningen nematode collection, The Netherlands (WaNeCo), were included for comparison (*viz*., *S. aberrans* (Whitehead, 1959b) Sher, 1961; *S. africanum* Smit, 1971; *S. brachyurus* (Steiner, 1938) Andrássy, 1958; *S. brevistyletum* Siddiqi, 1972; *S. cavenessi*; *S. clathricaudatum*; *S. conicephalum* Sivakumar & Selvasekaran, 1982; *S. erectum* Sivakumar & Khan, 1981; *S. labiatum* Siddiqi, 1972; *S. magniphasma* Sher, 1964; *S. naveum* Sivakumar & Khan, 1981; *S. truncatum* Sher, 1964; and *S. unum* Sher, 1964). Scanning electron microscopy (SEM) of selected specimens was performed as described by Steel *et al*. (2011).

### Molecular Characterisation

#### *DNA extraction and PCR amplification of the 28S rDNA and* COI *gene*

Following morphological identification, individual nematodes from temporary slides were picked and used for extraction of genomic DNA using a quick alkaline lysis protocol adapted from Schneider *et al*. (2015) (see Janssen *et al.*, 2016). PCRs were performed following the protocol of the D2-D3 expansion segment of the large sub-unit (LSU) rDNA and the Cytochrome c oxidase subunits 1 (*COI*) as described in Van den Berg *et al*. (2013). The primer sets D2A (5′-ACA AGT ACC GTG AGG GAA AGT TG-3′) and D3B (5′-TCG GAA GGA ACC AGC TAC TA-3′) were used for the amplification of the D2-D3 expansion regions of 28S rDNA gene. The Cytochrome c oxidase subunit 1 (*COI*) gene fragment was amplified using the primer sets JB3 (5′-TTT TTT GGG CAT CCT GAG GTT TAT-3′) and JB4 (5′-TAA AGA AAG AAC ATA ATG AAA ATG-3′).

PCR products were separated by electrophoresis on a 1% agarose gel and stained with ethidium bromide. PCR products were purified as described in the manufacturer’s instructions (Wizard® SV Gel and PCR Clean-Up System Kit, Promega) and sequenced by Macrogen (Europe) in both forward and reverse directions. Consensus sequences were assembled using GENEIOUS 9.15 (Biomatters; http://www.geneious.com) and deposited in the NCBI GenBank (Table 1).

#### Phylogenetic analysis

The D2-D3 of 28S rDNA and mt*COI* sequence generated in this study and sequences available for genus *Scutellonema* in the GenBank were aligned using MUSCLE (Edgar, 2004) with default settings. Outgroup taxa of each dataset were chosen based on previously published data (Van den Berg *et al.*, 2013). The best-fit models of DNA evolution were estimated using the program jModeltest 0.1.1 (Posada, 2008) under the Akaike information criterion (AIC). Bayesian phylogenetic analysis (BI) was done using MrBayes 3.2.6 (Huelsenbeck & Ronquist, 2001) for 5 × 10^6^ generations with a general time-reversible model with a gamma distribution for the remaining sites (GTR + I + G) for D2-D3 and *COI*. Two runs were performed for each analysis. After discarding burn-in samples and evaluating convergence, the remaining samples were used to generate a 50% majority rule consensus trees. Posterior probabilities (PP) were plotted and given on clades with *>*0.7 PP support. Pairwise divergences between taxa were computed as distance values and as percentage mean distance values based on the whole alignment, with adjustment for missing data using Geneious 9.15 (Kearse *et al.*, 2012). To test distinctiveness of putative species, generated trees were imported into Geneious where the species delimitation plugin (Masters *et al.*, 2011) was used to calculate Rosenberg’s P_AB_, which tests the probability for reciprocal monophyly of the clusters (Rosenberg, 2007).

## Results

### Morphological and Molecular Characterisation

Using morphological and molecular data, the following taxa from yam tubers and yam rhizosphere were identified: *S. bradys, S. cavenessi, S. clathricaudatum, Scutellonema* sp. D *sensu* Van den Berg *et al*. (2013), and two unknown species: *Scutellonema* sp. 1 and *Scutellonema* sp. 2. Ninety-nine sequences of *Scutellonema* (45 D2-D3 and 54 of *COI*) were generated from 45 populations. Of the 99 sequences, 87 were from nematodes obtained from yam tubers or yam rhizosphere and 12 from *Scutellonema* species collected from other crops (banana, carrot, maize and tomato) (Table 1).

The unknown species were considered different from all known species based on morphological differences, their unique phylogenetic position and molecular species delimitation. In addition, *S. brachyurus* was identified from banana, *S. cavenessi* from onion, and *S. paralabiatum* Siddiqi & Sharma, 1994 from banana, maize and onion rhizosphere (Table 1).

**Fig. 1.**
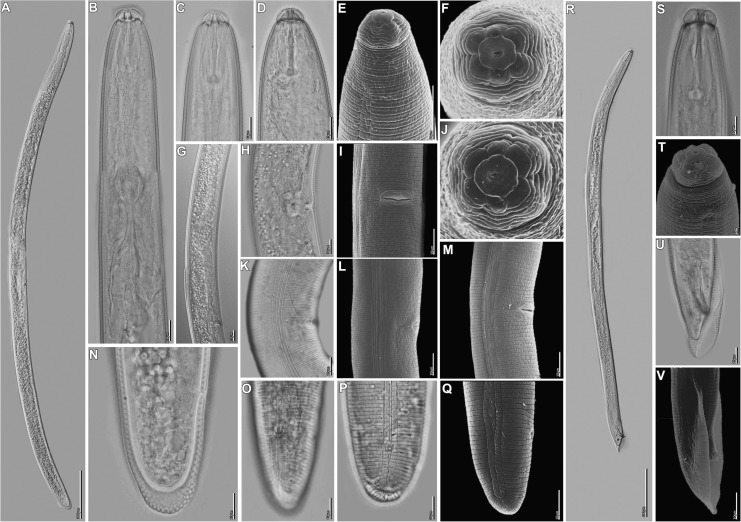
*Scutellonema bradys* (Steiner & LeHew, 1933) Andrássy, 1958. Light micrographs and scanning electron micrographs (SEM) of female (A-Q) and male (R-V). A: Entire body; B: Pharynx; C, D: Anterior end; E: Lateral view of female lip region (SEM); F, J: Face views of lip regions (SEM); G: Part of female reproductive system showing genital tract and functional spermathecal; H: Vulval region showing ‘vaginal glands’; I: Vulva (SEM); K: Vulval region showing lateral field; L, M: Vulval region showing lateral field (SEM); N: Tail; O-Q: Lateral field at scutellum (O, P: LM; Q: SEM); R: Male entire body; S: Male anterior end; S: Male lip region (SEM); U, V: Male tail (U: LM; V: SEM). (Scale bars: A, R = 100 *μ*m; B-Q, S-V = 10 *μ*m.)

**Fig. 2.**
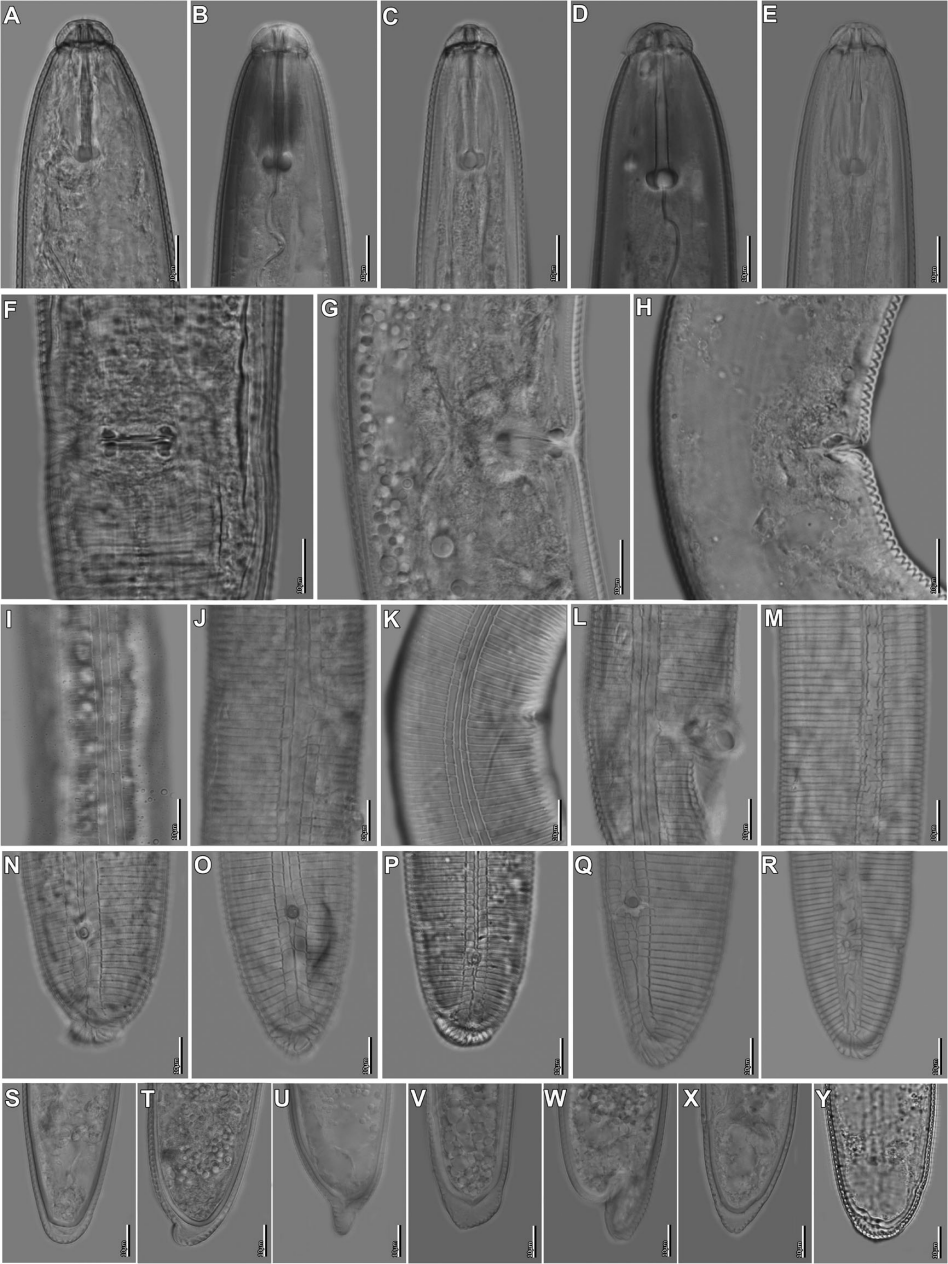
*Scutellonema bradys* (Steiner & LeHew, 1933) Andrássy, 1958, light micrographs of female showing morphological variation. A-E: Anterior end; F-H: ‘Vaginal glands’; I-M: Lateral field at mid-body; N-R: Lateral field at scutellum level; S-Y: Variations of tail end. (Scale bars = 10 *μ*m.)

Eleven populations used in this study were collected from yam rhizosphere, and yam tubers from separate locations in Nigeria (eight populations) and Ghana (three populations).

### Measurements

See Table 2.

**Table 2:** Morphometrics of female and male *Scutellonema bradys* from Ghana and Nigeria. All measurements are in *μ*m and in the form: mean ± s.d. (range)..

Character	Sample					
	YOS		3M2-5	3M10-1		3M17-8
	Female	Male	Female	Female	Male	Female
n	8	6	5	3	1	4
L	1003 ± 105 (804-1106)	937 ± 48 (894-1004)	1013 ± 72 (906-1109)	1112 ± 72(1055-1192)	864	1042 ± 100 (913-1146)
a	26.1 ± 2.9 (21.1-29.5)	30.8 ± 2.2 (28.9-34.5)	26.1 ± 0.95 (25.1-26.7)	25.3 ± 1.9 (23.5-27.2)	26.8	27.7 ± 5.3 (21.6-32.7)
b	8.1 ± 0.83 (6.3-9.0)	7.4 ± 0.85 (5.9-8.5)	8.8 ± 0.66 (8.3-9.8)	8.5 ± 0.16(8.4-8.6)	–	9.2 ± 2.0 (6.3-10.7)
b′	6.2 ± 0.6 (5.2-6.9)	5.6 ± 0.27 (5.2-5.9)	8.6 ± 0.15 (8.5-8.7)	8.8 ± 1.4 (7.9-9.8)	–	7.0 ± 0.88 (5.9-7.8)
c	35.8 ± 8.2 (27.2-53.9)	27.4 ± 2.3 (25.4-31.8)	30.2 ± 4 (26.9-36.4)	40.3 ± 3 (36.9-42.6)	30.9	29.3 ± 4.7 (23.7-35.3)
c′	1.0 ± 0.17(0.7-1.3)	1.6 ± 0.37 (0.91-2.0)	1.1 ± 0.21 (0.8-1.3)	1.0 ± 0.12(0.9-1.1)	1.2	1.3± 0.27(1.0-1.6)
o	23.6 ± 4.9 (17.6-32.4)	22.5 ± 2.2 (19.4-24.2)	28.6 ± 4.8 (24.4-35.5)	21.8 ± 7.6(14.4-29.6)	–	20.1 ± 12.2(11.5-28.8)
V	56.7 ± 1.9 (53.8-60.4)	–	55.9 ± 1.9 (53.5-57.8)	58.6 ± 0.97 (57.7-59.6)	–	56.2± 1.8(54.2-58.2)
Stylet	29.1 ± 1.6(27.2-32)	28.6 ± 1.6 (27.0-31)	26.6 ± 1.3 (25.5-28)	27.8 ± 0.76 (27.0-28.5)	25.5	28.3 ± 0.87 (27.5-29.5)
Conus	13.8 ± 1.4(12-16.5)	13.4 ± 2.6 (8.5-16.0)	12.5 ± 1.4(11.5-14.5)	12.8 ± 0.29(12.5-13.0)	12.0	13.9± 0.63(13.0-14.5)
Shaft and knobs	15.2 ± 1 (13.7-16.5)	15.2 ± 1.8 (13.5-18.5)	14.1 ± 0.63 (13.5-15.0)	15.0 ± 0.87(14.0-15.5)	13.5	14.4± 1.0(13.5-15.5)
Stylet width	2.8 ± 0.21 (2.6-3.2)	2.8 ± 0.13 (2.6-3)	–	2.3 ± 0.22 (2.1-2.4)	2.2	2.6 ± 0.33 (2.2-2.9)
m	47.5 ± 3.1 (42.1-51.4)	46.8 ± 7.7 (31.5-53.3)	46.9 ± 3.3 (45.1-51.8)	46.1 ± 1.8 (44.6-48.1)	47.1	49.1 ± 2.6 (46.4-51.8)
Stylet knob height	3.6 ± 0.2 (3.4-4.0)	3.5 ± 0.36 (2.8-3.7)	3.3 ± 0.14(3.1-3.4)	3.3 ± 0.0 (3.3-3.3)	–	3.7 ± 0.52 (3.4-4.1)
Stylet knob width	3.0 ± 0.43 (2.5-3.6)	2.9 ± 0.44 (2.2-3.3)	2.1 ± 0.21 (1.8-2.2)	2.9 ± 0.0 (2.9-2.9)	–	2.5 ± 0.71 (2.0-3.0)
Pharynx length	124 ± 9.0(112-140)	128 ± 13.8 (116-152)	116 ± 13.5 (92-124)	132 ± 13.9 (123-142)	–	120± 37(90-172)
Ant. endtomedian bulb valve	83 ± 4.1 (76-88)	83 ± 5.0 (75-88)	73 ± 6.5 (66-80)	80 ± 3 (78-82)	62	74 ± 11.5 (63-86)
Ant. endtopost end of gland	161 ± 11.5 (139-175)	168 ± 10.6 (156-181)	112 ± 6.6 (107-117)	131 ± 28.6 (111-152)	–	152 ± 23 (130-183)
Diam. at mid-body	39 ± 3.6 (34-44)	30.0 ± 1.2 (29.1-32)	40 ± 3.7 (38-44)	44 ± 3.7 (40-47)	32	39 ± 8.2 (31-50)
Diam. at anus	28.1 ± 2.4 (24.1-33.0)	23.2 ± 6.9 (19.3-37.0)	31 ± 4.6 (27.2-39.0)	26.8 ± 1.6 (25.2-28.3)	23.4	28.3 ± 6 (24.1-37)
Median bulb length	17.4 ± 1.7 (14.5-19.5)	17.1 ± 2.0 (15.0-20.0)	20.6 ± 10.8 (12.0-37.0)	20.0 ± 0.0 (20.0-20.0)	14.0	15.1 ± 4.0 (10.5-20.0)
Median bulb diam.	14.3 ± 1.1 (13.0-16.5)	13.2 ± 0.97 (12.0-14.5)	17.8 ± 5.5 (11.5-21.0)	17.5 ± 0.0 (17.5-17.5)	11.5	13.5 ± 2.7 (12.0-17.5)
Median bulb valve length	3.8 ± 0.38 (3.0-4.0)	3.6 ± 0.22 (3.5-4.0)	3.8 ± 0.29 (3.5-4.0)	4.0 ± 0.0 (4.0-4.0)		3.6 ± 0.63 (3.0-4.5)
Median bulb valve width	3.1 ± 0.32 (2.5-3.5)	2.6 ± 0.42 (2.0-3.0)	3.2 ± 0.29 (3.0-3.5)	2.0 ± 0.0 (2.0-2.0)		2.6 ± 0.63 (2-3.5)
Lipregiondiam.	11.7 ± 0.99(10.2-13.0)	11.9 ± 0.58 (11.1-12.8)	11.5 ± 0.64(10.7-12.1)	10.3 ± 3.9 (5.7-12.7)	10.2	12.1 ± 1.0 (11.0-13.4)
Lip region height	5.8 ± 0.7 (4.7-7.0)	5.3 ± 0.76 (4.8-6.9)	6.6 ± 0.56 (6.1-7.4)	8.9 ± 3.8 (6.6-13.3)	5.1	6.8 ± 0.37 (6.4-7.2)
Tail	28.9 ± 5.5 (20.5-37.0)	34.0 ± 3.7 (29.5-40.0)	34.0 ± 2.9 (31.0-38.0)	27.7 ± 2.0 (25.5-29.5)	28.0	36 ± 2.6 (33-39)
Scutellum length	4.6 ± 0.63 (3.3-5.3)	4.5 ± 0.51 (4.0-5.1)	4.5 ± 1.1 (3.1-5.8)	4.3 ± 0.4 (4.0-4.6)	3.5	4.7 ± 0.81 (3.8-5.4)
Scutellum width	4.1 ± 0.79 (2.9-5.3)	4.3 ± 0.74 (3.5-5.3)	4.2 ± 0.81 (3.0-4.9)	3.9 ± 0.07 (3.8-3.9)	3.4	4.6 ± 0.15 (4.4-4.7)
Spermatheca length	31 ± 15.6(18-66)	–	–	–	–	34 ± 12 (25.8-43)
Spermatheca diam.	22.6 ± 5.0 (12.5-28.1)	–	–	–	–	21.0 ± 1.1 (20.2-21.7)
Gonad anterior length	136 ± 60 (59-196)	136 ± 0.0 (136-136)	–	–	–	169 ± 0.0 (169-169)
Gonad posterior length	–	–	–	–	–	158 ± 0.0 (158-158)
Spicule length	–	33 ± 2.6 (31-38)	–	–	29.0	–
Ant. end to S-E/pharynx length	1.1 ± 0.08(1.1-1.3)	1.2 ± 0.08(1.1-1.3)	–	1.1 ± 0.0(1.1-1.1)	–	1.3 ± 0.03 (1.2-1.3)
n		4	5	3	5	5
L		1105 ± 50 (1055-1175)	935 ± 37 (907-995)	813 ± 54 (756-864)	1078 ± 96 (941-1176)	1006 ± 97 (891-1091)
a		23.2 ± 4.4 (19.0-29.5)	22.3 ± 1.7 (19.7-24.0)	23.0 ± 0.97 (22.4-24.1)	24.3 ± 2 (21.3-25.7)	24.9 ± 2.1 (22.5-27.9)
b		10.0 ± 0.64 (9.6-10.9)	8.2 ± 1.4 (5.7-8.9)	6.9 ± 0.95 (6.1-7.9)	6.9 ± 1.1 (5.9-8.5)	6.9 ± 1.3 (5.5-8.3)
b′		6.4 ± 1.8 (5.2-7.7)	4.9 ± 0.31 (4.6-5.2)	5.9 ± 0.83 (5.1-6.8)	5.8 ± 0.54 (5.4-6.7)	5.7 ± 0.58 (5.0-6.4)
c		32.6 ± 3.2 (29.3-36.7)	54.8 ± 18 (33.7-75.5)	35.3 ± 1.5 (33.6-36.4)	34.1 ± 0.82 (33.1-35.2)	30.8 ± 1.6 (29.0-33.3)
c′		1.0 ± 0.09 (0.95-1.1)	0.82 ± 0.42 (0.35-1.2)	0.95 ± 0.07 (0.88-1.0)	1.1 ± 0.09 (0.95-1.2)	1.3 ± 0.21 (1.1-1.5)
o		29.8 ± 2.1 (27.5-31.7)	–	23.1 ± 4.3 (20.0-26.1)	31.0 ± 0.73 (30.4-32.0)	24.4 ± 1.5 (23.3-25.4)
V		54.8 ± 1.8 (52.8-56.5)	–	57.3 ± 0.46 (56.8-57.6)	55.4 ± 1.7 (52.7-57.2)	–
Stylet		30 ± 1.0 (29.0-32.0)	28.7 ± 1.9 (27.0-31.0)	26.5 ± 1 (25.5-27.5)	31 ± 1.2 (29.5-32.0)	29.9 ± 1.1 (28.5-32.0)
Conus		13.3 ± 0.87(12.5-14.5)	14.2 ± 0.97 (13.0-15.5)	13.2 ± 2(11.0-15.0)	14.2 ± 1.4(12.5-16.0)	13.1 ± 1.7 (10.5-15.0)
Shaft and knobs		16.9 ± 0.63 (16.0-17.5)	14.5 ± 2(12.5-17.5)	13.3 ± 1 (12.5-14.5)	16.5 ± 0.94(15.5-17.5)	16.8 ± 0.91 (16.0-18.0)
Stylet width		2.8 ± 0.57 (2.3-3.4)	3.0 ± (3.0-3.0)	2.3 ± 0.0 (2.3-2.3)	2.9 ± 0.32 (2.7-3.3)	2.8 ± 0.52 (2.1-3.4)
m		44 ± 1.9 (41.7-46.0)	49.6 ± 4.2 (43.5-53.7)	49.5 ± 5.8 (43.1-54.5)	46.2 ± 3.3 (42.4-50.8)	43.7 ± 4.5 (36.8-47.6)
Stylet knob height		5.2 ± 0.95 (4.1-5.7)	3.1 ± 0.39 (2.8-3.7)	3.5 ± 0.0 (3.5-3.5)	3.7 ± 0.42 (3.3-4.1)	3.4 ± 0.15 (3.3-3.6)
Stylet knob width		3.5 ± 0.44 (3.0-3.7)	2.9 ± 0.23 (2.7-3.3)	2.6 ± 0.0 (2.6-2.6)	3.1 ± 0.13 (3.0-3.2)	2.7 ± 0.17 (2.5-2.9)
Pharynx length		111 ± 8.5 (100-120)	119 ± 32(102-175)	119 ± 8.8 (109-125)	160 ± 32(131-195)	147 ± 14.4 (132-165)
Ant. endtomedianbulbvalve		92 ± 15.9 (79-114)	69 ± 8.8 (61-79)	77 ± 7.6 (72-82)	103 ± 10.3 (90-117)	87 ± 16.5 (67-100)
Ant. end to post end of gland		182 ± 42(153-212)	193 ± 6 (187-199)	140 ± 16.8 (127-159)	186 ± 24(165-212)	176 ± 7.4(164-183)
Diam. at mid-body		49 ± 6.5 (40-56)	42 ± 3 (38-46)	35 ± 3.7 (31-39)	44 ± 3.3 (40-49)	40 ± 3.5 (35-45)
Diam. at anus		33 ± 4.6 (28-38)	26.3 ± 9.1 (15.2-36)	24.3 ± 2.6 (22.0-27.2)	29.3 ± 0.42 (28.6-29.7)	25.1 ± 2.2 (22.9-28)
Median bulb length		18.3 ± 1.3 (16.5-19.5)	16.3 ± 0.96 (15.5-17.5)	–	20.7 ± 3.4 (16.0-24.0)	14.8 ± 0.35 (14.5-15)
Median bulb diam.		17 ± 3.2(14.0-20.0)	15.9 ± 1 (15.0-17.0)	–	15.5 ± 1.9 (12.5-17.5)	12.5 ± 2.8 (10.5-14.5)
Median bulb valve length		4.5 ± 0.41 (4.0-5.0)	2.5 ± 0 (2.5-2.5)	–	4.6 ± 0.42 (4.0-5.0)	3.8 ± 0.35 (3.5-4)
Median bulb valve width		3.6 ± 0.48 (3.0-4.0)	2.0 ± 0 (2.0-2.0)	–	3.7 ± 0.27 (3.5-4.0)	2.8 ± 0.35 (2.5-3.0)
Lipregiondiam.		12.7± 0.38 (12.3-13.1)	11.2 ± 2.1 (7.8-13.7)	11.6 ± 0.58 (11.1-12.3)	12.0 ± 0.32(11.6-12.3)	11.2 ± 2.3 (7.2-13.1)
Lip region height		7.1 ± 0.38 (6.8-7.5)	6.1 ± 0.71 (4.9-6.7)	6.3 ± 1.2 (5.3-7.6)	6.2 ± 1.0 (5.3-7.4)	5.4 ± 0.95 (3.7-5.9)
Tail		34 ± 1.8 (32-36)	18.8 ± 7 (12.5-29.5)	23.0 ± 0.87 (22.5-24.0)	32 ± 2.9 (28.0-34.0)	33 ± 3.4 (29.5-38)
Scutellum length		5.1 ± 0.83 (4.2-5.9)	3.8 ± 1.1 (2.8-5.6)	4.1 ± 0.89 (3.4-5.1)	5.4 ± 0.61 (4.6-6.0)	5.0 ± 0.81 (4-6.2)
Scutellum width		4.8 ± 0.73 (3.9-5.7)	3.7 ± 0.87 (3.0-5.2)	4.2 ± 0.89 (3.6-4.8)	5.5 ± 0.24 (5.3-5.8)	5.0 ± 1 (3.8-6.4)
Spermatheca length		34 ± 12.8 (24.7-43)	–	28.6 ± 2.1 (27.1-30.0)	33 ± 7.9 (24.7-43)	–
Spermatheca diam.		16.9 ± 6.8 (12.2-21.7)	–	21.7 ± 1.1 (20.9-22.5)	18.2 ± 5.5(12.2-22.4)	–
Gonad anterior length		–	–	109 ± 0 (109-109)	–	–
Gonad posterior length		158 ± (158-158)	–	–	109 ± 43 (84-158)	–
Spicule length		–	37 ± 6.2 (31-44)	–	–	32 ± 2.6 (28.9-35)
Ant. end to S-E/pharynx length		1.3 ± 0.03 (1.3-1.3)	–	1.1 ± 0.06(1.0-1.1)	0.92 ± 0.18(0.74-1.2)	0.94 ± 0.17 (0.82-1.1)
n	1	6	10	5	10	10
L	719	815 ± 78 (740-928)	995 ± 110 (809-1129)	1019 ± 81 (892-1084)	1100 ± 123 (921-1315)	957 ± 118 (790-1123)
a	19.0	18.6 ± 1.3 (17.5-21.1)	21.1 ± 3.5 (17.2-27.8)	24.3 ± 2 (21.6-27.0)	27.9 ± 4.5 (21.9-36)	26.9 ± 2.4 (22.6-30.4)
b	8.7	7.5± 0.97 (6.2-8.6)	8.0 ± 0.87 (6.9-9.6)	7.2 ± 0.97 (5.5-7.7)	9.0 ± 0.83 (8.1-10.6)	7.8 ± 0.39 (7.4-8.7)
b′	6.2	5.6 ± 0.45 (5.0-6.3)	6.5 ± 0.87 (5.2-8.0)	6.1 ± 0.97 (4.7-6.9)	7.1 ± 0.67 (6.4-8.4)	6.3 ± 0.66 (5.2-7.3)
c	23.2	30.0 ± 5.3 (24.0-37.9)	31.5 ± 4.7 (25.6-41.2)	32.8 ± 6.7 (25.0-39.3)	34.9 ± 3.4 (30.4-42)	30.8 ± 5.3 (21.9-37.4)
c′	1.0	0.94 ± 0.1 (0.82-1.1)	1.0 ± 0.11 (0.88-1.3)	1.4 ± 0.16(1.1-1.6)	1.2 ± 0.07(1.0-1.2)	1.5 ± 0.13 (1.2-1.7)
o	26.6	18.3 ± 5.1 (15.3-24.2)	24.4 ± 5.9 (14.8-30.0)	27.4 ± 4.4 (24.2-30.5)	26.3 ± 4.8 (20.0-30.0)	28.2 ± 2.7 (25.7-31.1)
V	55.0	56.8 ± 1.4 (55.2-58.8)	55.1 ± 2.4 (50.6-57.8)	–	54.7 ± 2.4 (50.8-58)	–
Stylet	26.2	27.0 ± 1.7 (24.2-28.5)	29.8 ± 1.4 (27.0-32)	29.5 ± 3.3 (25.5-33)	28.1 ± 1.8 (25.0-30.0)	26.3 ± 1.8 (24.0-30.0)
Conus	13.5	11.7 ± 1.4(10.0-13)	13.4 ± 1.4(11.5-16.0)	12.7 ± 3 (9.5-16.0)	12.3 ± 0.98 (11.0-14.0)	11.5 ± 2.5 (9.0-15.5)
Shaft and knobs	12.7	15.3 ± 1.1 (14.0-16.5)	16.4 ± 1.1 (14.0-17.6)	16.8 ± 1 (15.5-18.0)	15.8 ± 1.3 (13.5-18)	14.8 ± 1.6 (11.5-17.5)
Stylet width	2.1	2.6 ± 0.61 (2.0-3.5)	3.0 ± 0.39 (2.5-3.5)	2.9 ± 0.31 (2.6-3.4)	2.4 ± 0.5 (1.6-3.0)	2.4 ± 0.45 (1.6-3.0)
m	51.5	43.4 ± 3.5 (38.5-48.1)	44.8 ± 3.7 (41.6-53.3)	42.6 ± 6 (35.2-48.5)	43.7 ± 2.3 (40-46.7)	43.4 ± 7.4 (35.2-57.4)
Stylet knob height	2.8	3.6 ± 0.17 (3.3-3.7)	3.6 ± 0.84 (2.8-5.7)	3.4 ± 0.7 (2.8-4.1)	3.2 ± 0.27 (2.8-3.4)	3.2 ± 0.44 (2.6-4.0)
Stylet knob width	2.2	2.4 ± 0.33 (1.9-2.8)	3.3 ± 0.45 (2.7-3.9)	2.7 ± 0.19(2.5-2.9)	2.5 ± 0.49 (2.0-3.0)	2.6 ± 0.47 (2.0-3.1)
Pharynx length	83	110 ± 12.8 (94-124)	125 ± 10.5 (109-142)	144 ± 13.5 (128-163)	123 ± 10.7 (104-136)	122 ± 12.5 (104-136)
Ant. endtomedianbulbvalve	60	81 ± 4.2 (76-87)	87 ± 7.9 (79-105)	91 ± 10.5 (75-103)	80 ± 6.6 (70-91)	84 ± 7.0 (70-91)
Ant. end to post end of gland	117	147 ± 7.6(141-159)	152 ± 12.5 (133-170)	169 ± 15.6 (152-190)	153 ± 16.9 (128-170)	154 ± 24.3 (128-215)
Diam. at mid-body	38	44 ± 3.8 (40-51)	48 ± 5.7 (41-56)	42 ± 2.4 (39-46)	40 ± 3.9 (35-46)	36 ± 3.2 (29.2-39)
Diam. at anus	30	29.3 ± 1.5 (26.7-31)	31 ± 3.3 (25.9-38)	23.4 ± 4.1 (19.6-29.8)	27.5 ± 3.5 (21.7-32)	21.3 ± 3.4(18.0-30.0)
Median bulb length	13.5	14.6 ± 0.63 (14.0-15.5)	17.4 ± 3.4(14.5-24.5)	15.0 ± 0.71 (14.5-15.5)	15.0 ± 1.6 (13.0-17.5)	14.5 ± 1.1 (13.0-15.5)
Median bulb diam.	14	12.3 ± 1.8 (10.5-14.0)	15.0 ± 3 (12.0-20.0)	10.5 ± 0 (10.5-10.5)	13.0 ± 2.3 (10.5-16.5)	11.1 ± 0.79(10.5-12.5)
Median bulb valve length	3.5	3.3 ± 0.29 (3.0-3.5)	4.1 ± 0.52 (3.5-5.0)	3.8 ± 0.35 (3.5-4.0)	3.8 ± 0.35 (3.0-4.0)	3.4 ± 0.35 (3.0-4.0)
Median bulb valve width	3	2.8 ± 0.65 (2.0-3.5)	3.6 ± 0.35 (3.0-4.0)	2.5 ± 0 (2.5-2.5)	3.1 ± 0.52 (2.5-4.0)	2.9 ± 0.48 (2.5-3.5)
Lip region diam.	11.2	10.7 ± 0.71 (10.1-11.7)	12.0 ± 0.65 (11.2-13.1)	10.6 ± 3.1 (7.1-14.3)	11.6 ± 0.98 (10.2-13)	10.9 ± 1.4 (7.4-12.6)
Lip region height	5.6	6.0 ± 0.83 (5.1-7.0)	5.5 ± 1.3 (4.2-7.5)	5.0 ± 1.2 (3.6-6.3)	5.7 ± 0.77 (4.6-7.0)	5.4 ± 0.85 (4.0-7.4)
Tail	31	27.5 ± 2.3 (24.5-31)	32 ± 4.1 (26-37)	32 ± 5 (27.5-40)	32 ± 4.6 (26.5-38)	31 ± 3.5 (28.0-37.0)
Scutellum length	4.3	4.5 ± 0.69 (3.8-5.5)	4.8 ± 0.48 (4.1-5.7)	5.0 ± 0.83 (4.1-6.2)	4.2 ± 0.49 (3.5-5.0)	4.1 ± 0.79 (2.9-5.1)
Scutellum width	4.1	4.5 ± 0.67 (3.7-5.5)	4.7 ± 0.39 (4.0-5.2)	5.0 ± 1.1 (3.9-6.4)	4.0 ± 0.51 (3.0-5.0)	4.0 ± 0.9 (2.8-5.3)
Spermatheca length	39	26.4 ± 15.8 (12.6-40.0)	29 ± 8.8 (18.5-40)	–	29.7 ± 8.4(17.1-34)	–
Spermatheca diam.	27.9	17.4 ± 9.7 (9.0-25.9)	21 ± 5 (12.5-25.9)	–	21.3 ± 5.6(12.9-24.4)	–
Gonad anterior length	75	73 ± 0 (73-73)	36 ± 0 (36-36)	–	141 ± 0.08 (141-141)	–
Gonad posterior length	60	90 ± 0 (90-90)	59 ± 25.2 (41-90)	–	115 ± 0.13 (115-115)	–
Spicule length	–	–	–	33 ± 2.1 (29.7-35)	–	33 ± 3.7 (24.3-37)
Ant. end to S-E/pharynx length	1.1	1.1 ± 0.09 (1.0-1.2)	1.1 ± 0.07 (1-1.2)	–	1.1 ± 0.1 (1.0-1.3)	0.97 ± 0.07 (0.86-1)

S-E = secretory/excretory pore position.

### Description

#### Female

Body straight to slightly curved ventrally after fixation.

Lateral field areolated at anterior portion of body and at scutellum level, smooth to partially areolated at mid-body. Lip region, hemispherical, offset by slight to deep constriction with seven (5-9) annuli and without longitudinal striations on basal lip annulus (observation from SEM). Labial disc rounded with small amphidial openings laterally. Stylet robust with knobs round to oval at base and in some cases with irregular anterior margin. Conus often shorter than shaft and knobs combined, m = 45.8 (38.5-54.5)%. Median bulb spherical to oblong. Pharyngeal gland lobes overlapping intestine dorsally. Excretory pore often located at posterior level of pharyngeal gland lobe, 137 (94-159) *μ*m from anterior end. Hemizonid 0-3 annuli anterior to excretory pore. Spermatheca rounded to oval, filled with sperm cells. Often, with conspicuous ‘vaginal glands’ arranged around vulva (four in ventral view and two in lateral view). Epiptygmata often absent, otherwise small and appearing double. Scutellum moderate sized with rounded shape, located opposite or slightly anterior or posterior to anus. Tail variable in shape, often tapering gradually with rounded end and striated terminus. Tail 1.1 (0.7-1.6) anal body diam. long and with 20 (13-25) annuli.

#### Male

Similar to female except for reproductive structures, bursa relatively narrow, not lobe-shaped with abrupt narrowing.

### Diagnosis and Relationships

*Scutellonema bradys* is characterised by a straight to slightly ventrally curved female body. Lip region offset by a constriction with seven (5-9) lip annuli and lacking longitudinal striae on the basal lip annulus. Lateral field areolated at scutellum level. ‘Vaginal glands’ often present and well developed. Spermatheca present and filled with sperm cells.

*Scutellonema bradys* is similar to *S. cavenessi* from which it can be distinguished by its general habitus (slightly curved *vs* C-shaped), larger submedian lips, epiptygmata absent or very small *vs* long and protruding, and ‘vaginal glands’ conspicuous and very well developed *vs* not very well developed. Bursa relatively narrow *vs* lobe-shaped with abrupt narrowing.

**Fig. 3.**
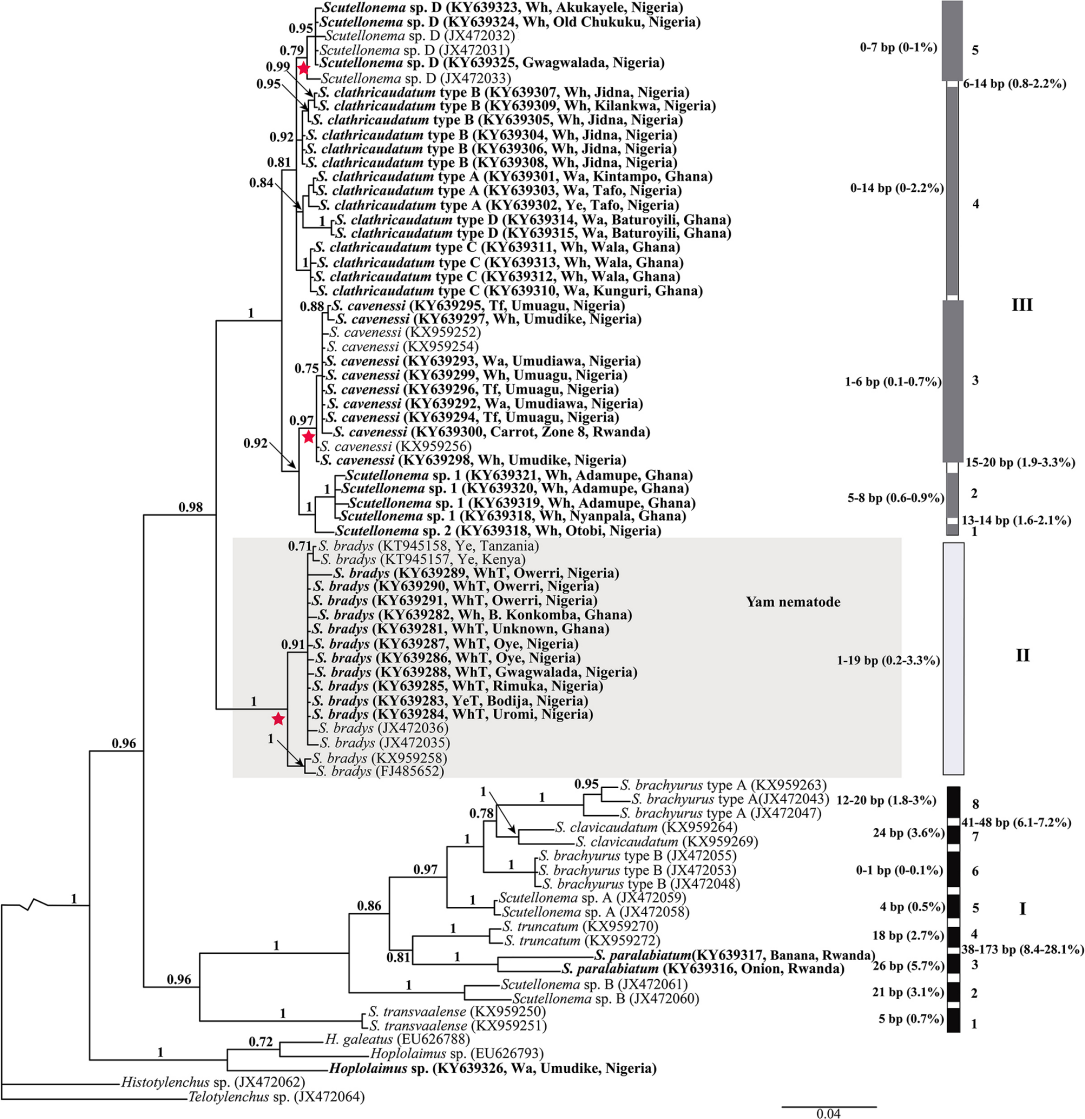
Phylogenetic relationships within *Scutellonema*. Bayesian 50% majority rule consensus tree as inferred from the analysis of the D2-D3 expansion segments of 28S rDNA sequence alignment under a GTR + I model. Newly obtained sequences are indicated in bold. Posterior probabilities equal or more than 0.7 are given. Intraspecific variation of a clade indicated by a bar is given to the left of the bar, nucleotide differences between sister clades is provided right to the bars. Thick bars are clades that are supported in both analyses and by significant Rosenberg’s species delimitation probabilities. Species that are supported as distinct taxonomic identities with significant Rosenberg’s probabilities are indicated by a star.

**Fig. 4.**
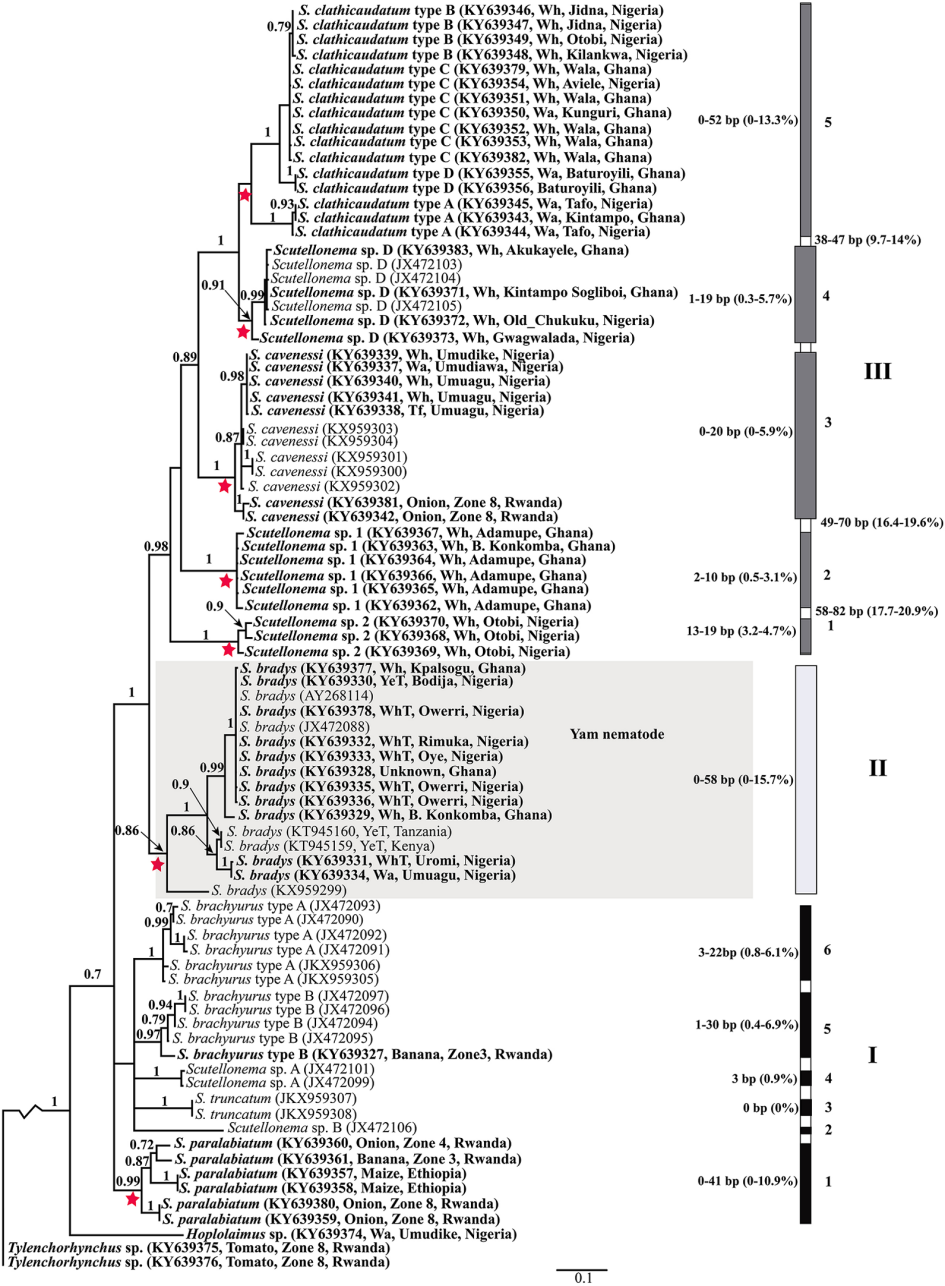
Phylogenetic relationships within *Scutellonema*. Bayesian 50% majority rule consensus tree as inferred from the analysis of the *COI* mtDNA sequence alignment under a GTR + I + G model. Newly obtained sequences are indicated in bold. Posterior probabilities equal or more than 0.7 are given. Intraspecific variation of a clade indicated by a bar is given to the left of the bar, nucleotide differences between sister clades is provided right to the bars. Thick bars are clades that are supported in both analyses and by significant Rosenberg’s species delimitation probabilities. Species that are supported as distinct taxonomic identities with significant Rosenberg’s probabilities are indicated by a star.

*Scutellonema bradys* sequences form, based on both D2-D3 and *COI*, a maximally supported clade (Clade II that is sister to Clade III). (Figs 3, 4). However, the intraspecific molecular variation for *S. bradys* is very high, 1-19 bp (0.2-3.3%) and 0-58 bp (0.0-15.7%) for D2-D3 and *COI* respectively. Species delimitation strongly supports reciprocal monophyly of *S. bradys* in respect to its sister clade (Rosenberg’s P_AB:_ 5.4E−16 and 2.2E−16 based on D2-D3 and *COI* tree topologies, respectively). The interspecific differences between *S. bradys* and *S. cavenessi* were 33-54 bp (6.0-8.8%) and 59-80 bp (16.8-21.8%) for the D2-D3 and *COI*, respectively.

### Remarks

*Scutellonema bradys* was the only *Scutellonema* species retrieved from yam tubers. Adults from tubers are relatively large compared with those from the rhizosphere (Table 2).

**Fig. 5.**
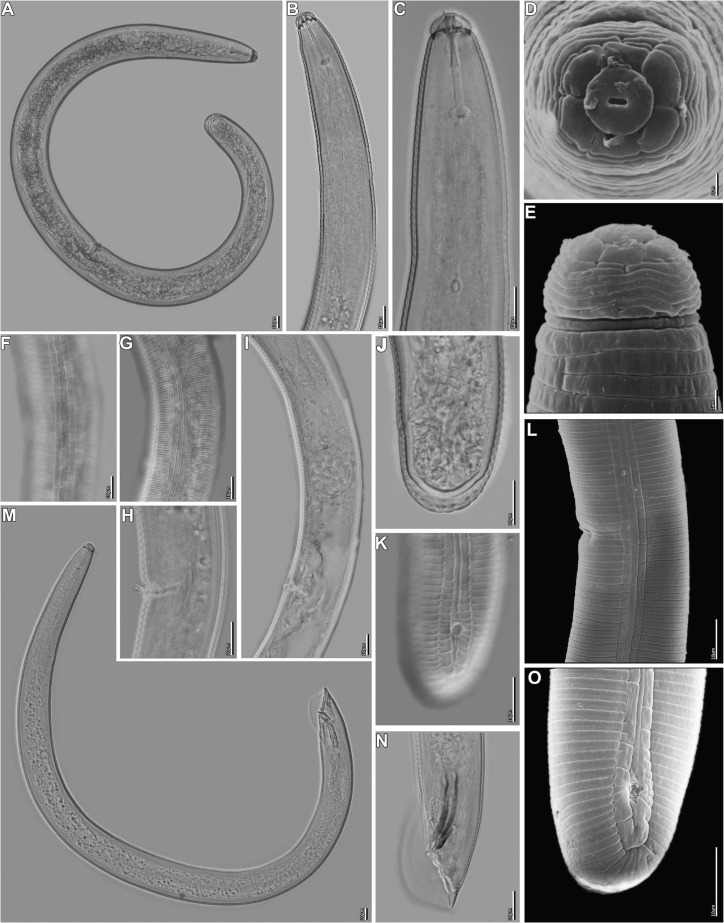
*Scutellonema cavenessi* Sher, 1964. Light micrographs and scanning electron micrographs (SEM) of female (A-L, O) and male (M, N). A: Entire body; B: Pharynx; C: Anterior end; D: Face view of lip region (SEM); E: Lateral view of lip region (SEM); F, G, L: lateral field at vulval region (F, G: LM; L: SEM); H: Vulval region showing epiptygmata; I: Part of female reproductive system showing functional spermatheca, J: Tail; K, O: Lateral field at scutellum (K: LM; O: SEM); M: Male entire body; N: Male tail. (Scale bars: A-C, F-O = 10 *μ*m; D, E = 1 *μ*m.)

Four populations were obtained from yam rhizosphere from separate locations in Nigeria.

### Measurements

See Table 3.

**Table 3:** Morphometrics of female and male of *Scutellonema cavenessi* from Nigeria. All measurements are in *μ*m and in the form: mean ± s.d. (range).

Character	Sample							
	2NS13-1		2NS15-9		2NS16-1		2NS16-5	
	Female	Male	Female	Male	Female	Male	Female	Male
n	3	1	1	1	7	1	3	3
L	773 ± 28.3 (755-805)	688	609	586	691 ± 53 (587-734)	674	756 ± 23.8 (733-781)	605 ± 74 (532-679)
a	21.5 ± 1.5 (20.0-23.0)	20.5	19.4	16.8	21.1 ± 2.9 (18.2-26.5)	20.2	19.5 ± 1.2(18.2-20.3)	20.0 ± 1.2(18.6-20.8)
b	7.7 ± 0.52 (7.1-8.1)	6.0	6.8	6.5	7.3 ± 1.3 (5.9-9.9)	7.7	8.6 ± 1.6 (7.6-10.4)	6.9 ± 1.2(5.8-8.2)
b′	6.0 ± 0.18 (5.9-6.2)	5.1	5.3	5.0	5.9 ± 1.1 (4.8-8.2)	6.1	7.2 ± 2.5 (5.7-10.2)	5.1 ± 1.0 (4.3-6.2)
c	43.7 ± 0.88 (43.2-44.7)	27.0	30.5	30.8	36.8 ± 6.9 (29.3-50.3)	26.4	35.9 ± 3.4 (33.3-39.7)	26.0 ± 2.3 (23.6-28.1)
c′	0.72 ± 0.11 (0.65-0.85)	1.3	0.83	1.1	0.76 ± 0.15 (0.52-0.95)	1.1	0.75 ± 0.06 (0.7-0.82)	1.2 ± 0.22(1.1-1.5)
o	18.2 ± 4.1 (15.7-23.0)	19.1	28.9	24.2	26.2 ± 7.7(17.8-40.8)	34.0	12.6 ± 0.0(12.6-12.6)	29.4 ± 2.0 (27.6-31.5)
V	56.7 ± 1.2(55.8-58.1)	–	56	–	58.0 ± 1.6 (55.6-59.8)	–	56.3 ± 1.5 (55-57.9)	–
Stylet	24.3 ± 0.76 (23.5-25.0)	24.5	23.0	22.5	24.1 ± 0.69 (23.5-25.5)	23.0	24.8 ± 1.5 (23.5-26.5)	24.0 ± 1.3 (23.0-25.5)
Conus	12.0 ± 1.0 (11.0-13.0)	14.0	10.5	10.5	10.9 ± 1.1 (9.5-13.0)	10.0	10.5 ± 1.5 (9.0-12.0)	11.7 ± 1.2(11.0-13.0)
Shaft and knobs	12.3 ± 0.76(11.5-13.0)	10.5	12.5	12.0	13.2 ± 0.81 (12.5-14.5)	13.0	14.3 ± 0.29 (14.0-14.5)	12.3 ± 0.29(12.0-12.5)
Stylet width	2.1 ± 0.11 (2.0-2.2)	1.9	2.2	2.0	2.2 ± 0.33(1.9-2.9)	1.9	2.3 ± 0.04 (2.3-2.4)	2.0 ± 0.08 (1.9-2.0)
m	49.3 ± 3.3 (46.8-53.1)	57.1	45.7	46.7	45.2 ± 3.6 (39.6-51)	43.5	42.1 ± 3.5 (38.3-45.3)	48.5 ± 2.2(46.8-51.0)
Stylet knob height	2.9 ± 0.16 (2.8-3.0)	3.2	3.2	2.5	2.9 ± 0.37 (2.6-3.6)	2.3	3.4 ± 0.52 (2.9-4.0)	3.5 ± 0.69 (3.0-4.0)
Stylet knob width	2.7 ± 0.27 (2.5-2.9)	2.1	2.2	2.0	2.5 ± 0.34(1.9-2.8)	1.8	2.9 ± 0.6 (2.2-3.4)	2.1 ± 0.0 (2.1-2.1)
Pharynx length	101 ± 5.1 (97-107)	115	90	89	97 ± 20.2 (67-124)	88	90 ± 13.3 (75-100)	89 ± 12.9 (81-104)
Ant. end to median bulb valve	70 ± 3.3 (68-74)	69	60	63	66 ± 11.1 (47-79)	65	55 ± 10.5 (45-66)	66 ± 3.6 (64-70)
Ant. end to post. end of gland	128 ± 2.5 (125-130)	136	116	116	121 ± 25.5 (80-152)	110	111 ± 30 (77-132)	121 ± 10.8 (109-130)
Diam. at mid-body	36 ± 1.5 (35-38)	34	31	35	33 ± 4.4 (27.5-40)	33	39 ± 3.5 (37-43)	30.0 ± 5.5 (25.6-36)
Diam. at anus	24.7 ± 3.6 (20.6-27.1)	19.1	24.0	17.1	25.6 ± 2.3 (22.6-28.1)	24.1	28.3 ± 2.3 (27.0-31)	19.4 ± 4.8 (15.2-24.7)
Median bulb length	15.5 ± 0.5 (15.0-16.0)	15.0	12.0	13.5	15.0 ± 1.8 (12.0-17.5)	–	14.5 ± 1.4(13.5-15.5)	13.3 ± 2.0 (11.0-14.5)
Median bulb diam.	12.8 ± 1.3 (11.5-14.0)	11.5	11.5	11.5	11.9 ± 1.1 (10.0-13.5)	–	13.3 ± 1.8 (12-14.5)	10.7 ± 1.3 (9.5-12.0)
Median bulb valve length	4.0 ± 0 (4.0-4.0)	2.5	3.5	3.5	3.4 ± 0.24 (3.0-3.5)	–	3.8 ± 0.35 (3.5-4.0)	3.2 ± 0.29 (3.0-3.5)
Median bulb valve width	2.8 ± 0.35 (2.5-3.0)	2.0	3.0	3.0	2.6 ± 0.24 (2.5-3.0)	–	2.8 ± 0.35 (2.5-3.0)	2.3 ± 0.76 (1.5-3.0)
Lipregiondiam.	10.8 ± 0.31 (10.5-11.0)	10.3	10.2	9.9	9.8 ± 0.67 (9.0-10.8)	8.8	10.1 ± 0.84 (9.6-11.1)	9.5 ± 0.83 (9.0-10.4)
Lip region height	6.4 ± 0.8 (5.5-6.9)	6.1	5.5	4.8	5.4 ± 0.61 (4.7-6.5)	4.7	5.9 ± 0.57 (5.2-6.3)	5.3 ± 1.2 (4.4-6.7)
Tail	17.7 ± 0.29(17.5-18.0)	25.5	20.0	19.0	19.1 ± 2.8 (14.5-23.5)	25.5	21.2 ± 1.9 (19.0-22.5)	23.3 ± 2.4 (21.5-26.0)
Scutellum length	4.6 ± 0.32 (4.4-5.0)	3.9	4.6	4.0	4.5 ± 0.35 (4.0-5.0)	4.1	5.1 ± 0.29 (5.0-5.5)	3.7 ± 0.23 (3.5-3.9)
Scutellum width	4.0 ± 0.24 (3.8-4.2)	3.3	4.1	3.2	4.1 ± 0.38(3.6-4.6)	3.4	4.8 ± 0.32(4.5-5.1)	3.3 ± 0.29 (3.1-3.6)
Spermatheca length	26.6 ± 4.4 (22.1-31)	–	17.8	–	17.8 ± 3.0(14.1-23.0)	–	–	–
Spermatheca diam.	20.8 ± 0.37 (20.5-21.2)	–	16.6	–	15.0 ± 2.6 (13.4-20.2)	–	–	–
Gonad anterior length	82 ± 10 (72-92)	–	89	–	72 ± 8.7 (62-83)	109	–	119 ± 0.0(119-119)
Gonad posterior length	85 ± 23 (69-102)	–	95	–	83 ± 13.7 (68-101)	–	–	–
Spicule length	–	37	–	30.0	–	33	–	29.3 ± 2.5 (26.5-31)
Ant. end to S-E/pharynx length	1.1 ± 0.0 (1.1-1.1)	–	–	0.9	–	1.0	–	–

S-E = secretory/excretory pore position.

### Description

#### Female

Body curved ventrally, inverted comma to C-shaped after fixation and tapering slightly towards anterior end. Cuticle at mid-body with annuli 2.1 *μ*m wide. Lateral field areolated at anterior portion of body and at scutellum level, smooth to partially areolated at mid-body, comprising one-fifth diam. of mid-body. Lip region, hemispherical, offset by slight to deep constriction with seven (5-8) annuli and lacking longitudinal striations on basal lip annulus (observation from SEM). Labial disc rounded with small amphidial openings laterally. Stylet well developed with knobs oval at base and slightly indented anteriorly. Conus often shorter than shaft and knobs combined, m = 45.5 (38.3-53.1)%. Median bulb spherical to oblong. Pharyngeal gland lobe overlapping intestine dorsally. Excretory pore often located at pharyngeal gland lobe level, 104 (83-129) *μ*m from anterior end. Hemizonid immediately anterior to excretory pore, 1-2 annuli long. Spermatheca rounded and filled with sperm cells. Vagina with non-developed to well-developed ‘vaginal glands’ arranged around vulva (seen as four in ventral view and two in lateral view). Epiptygmata often present and single, double in some rare cases. Scutellum rounded, moderate to large in size, located at level of anus. Tail with rounded end and striated terminus, slightly ventrally curved. Tail 0.75 (0.52-0.95) anal body diam. long and with 13 (8-17) annuli.

#### Male

Similar to female except for reproductive structures, with wide and broadly enveloping bursa.

### Diagnosis and Relationships

*Scutellonema cavenessi* is similar to *Scutellonema* sp. D *sensu* Van den Berg *et al*. (2013), *Scutellonema* sp. 1, *Scutellonema* sp. 2, *S. clathricaudatum* and *S. bradys* with respect to the lack of striation at the basal lip annulus. Morphologically, it is distinguished from *S. clathricaudatum* by the presence of the spermatheca and males. From *S. bradys, S. cavenessi* is distinguished by its relatively smaller size 716 (587-805) *vs* 1007 (719-1315) *μ*m, the general habitus (C-shaped *vs* slightly curved), shorter stylet of 24.3 (23.0-26.5) *vs* 28.5 (24.2-32.0) *μ*m, ‘vaginal glands’ often not developed *vs* well developed, presence of protruding epiptygmata *vs* absent to very small epiptygmata. Males of *S. cavenessi* are distinguished by broad bursa *vs* narrow bursa in *S. bradys*. Based on its size, *S. cavenessi* comes closer to *Scutellonema* sp. D, *Scutellonema* sp. 1 and *Scutellonema* sp. 2 from which it can be distinguished by having a well-developed spermatheca and a short and rounded tail (19.3 (14.5-23.5) *μ*m; c = 37.7 (29.3-50.0); c′ = 0.77 (0.52-0.95)). *Scutellonema cavenessi* sequences formed a highly supported clade (PP = 0.97) with an intraspecific variation of 1-6 bp (0.1-0.7%) and 0-20 bp (0-5.9%) for D2-D3 and *COI* respectively (Figs 3, 4). Molecular divergence between *S. cavenessi* and its sister taxon according to D2-D3, *Scutellonema* sp. 1 and *Scutellonema* sp. 2 is 13-14 bp (1.6-2.1%) (Fig. 3) and according to *COI*, 49-70 bp (16.4-19.6%) and 69-81 bp (18.5-21.4%) for *Scutellonema* sp. 1 and *Scutellonema* sp. 2 respectively (Fig. 4).

The species identity of *S. cavenessi* was also supported by significant Rosenberg’s P_AB_ values for both D2-D3 (P_AB_:2.0E−5) and *COI* (P_AB_:7E−11) (Figs 3, 4).

**Fig. 6.**
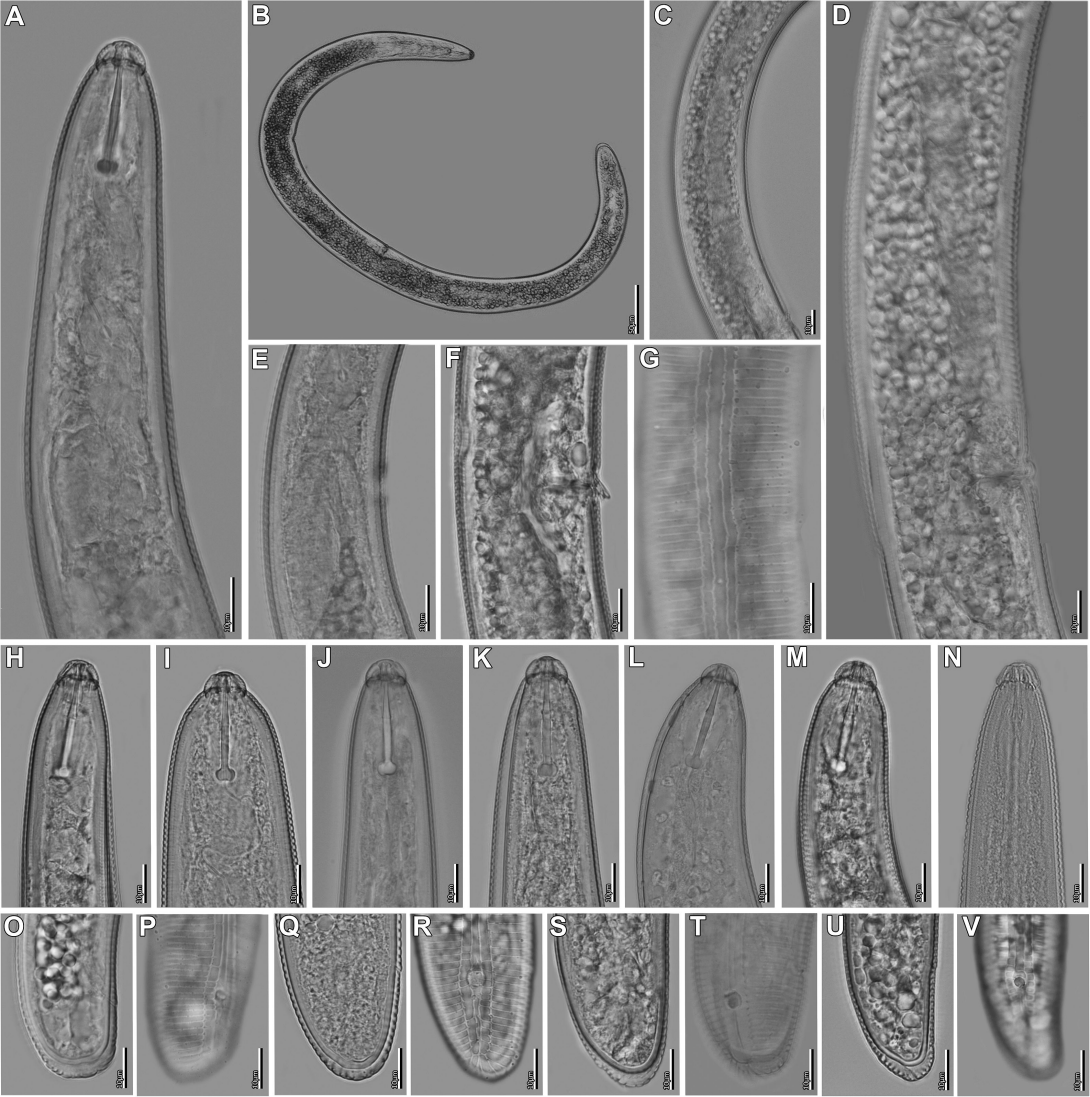
*Scutellonema clathricaudatum* (Whitehead, 1959). Female. A: Pharynx; B: Entire body; C, D: Reproductive system showing genital track; E: Pharynx base; Lateral field at mid-body; F: Vulval region showing epiptygmata; G: Lateral field at mid-body; H-N: Lip region; O-V: Variation in tail end. (Scale bars: A, C-V = 10 *μ*m; B = 50 *μ*m.)

Fifteen populations were analysed, all collected from yam rhizosphere from separate locations in Nigeria and Ghana.

### Measurements

See Tables 4–6.

**Table 4:** Morphometrics of female of *Scutellonema clathricaudatum* type A and S. clathricaudatum type D from Ghana and Nigeria. All measurements are in *μm* and in the form: mean ± s.d. (range).

Character	Sample			
	*S. clathricaudatum* type A		*S. clathricaudatum* type D	
	2NS35-5	2NS35-9	L17	L28
n	1	5	4	3
L	800	826 ± 40 (784-888)	648 ± 91 (512-710)	822 ± 112(699-919)
a	24.3	24.6 ± 3.6(18.8-28.2)	20.4 ± 3.3(16.5-24.5)	20.5 ± 1.2(19.3-21.6)
b	9.4	7.8 ± 1.4 (6.8-9.8)	7.4 ± 1.4 (5.7-8.9)	8.8 ± 1.1 (7.6-9.7)
b′	8.6	6.7 ± 1.5 (5.3-8.8)	5.6 ± 0.95 (4.7-6.6)	6.9 ± 1.5 (5.9-8.0)
c	50.0	45.3 ± 5.3 (37.8-50.9)	39 ± 7.0 (30.1-44.7)	26.2 ± 3.9 (23.7-30.6)
c′	0.68	0.77 ± 0.12(0.7-0.98)	0.71 ± 0.04 (0.67-0.76)	1.2 ± 0.11 (1.1-1.3)
o	–	27.1 ± 1.8 (25.2-28.7)	23.9 ± 1.4 (22.5-25.2)	26.3 ± 2.4 (24.6-27.9)
V	55.6	57.1 ± 1.5 (54.8-58.8)	54.2 ± 2.7 (52.2-57.3)	54.7 ± 1.3 (53.4-56.1)
Stylet	27.0	26.7 ± 1.3 (25.5-28.5)	25.9 ± 1.4 (24.5-27.5)	27.7 ± 1.3 (26.5-29.0)
Conus	11.5	11.2 ± 1.3 (10.0-13.0)	11.3 ± 1.0 (10.0-12.5)	12.7 ± 1.3 (11.5-14.0)
Shaft and knobs	15.5	15.5 ± 0.71 (14.5-16.5)	14.6 ± 0.75 (13.5-15.0)	15 ± 2.2(12.5-16.5)
Stylet width	2.9	2.4 ± 0.19(2.3-2.7)	2.0 ± 0.28(1.6-2.3)	2.4 ± 0.45 (2.1-2.7)
m	42.6	41.9 ± 3.3 (37.7-45.6)	43.4 ± 2.5 (40.0-45.5)	45.9 ± 6.0 (41.8-52.8)
Stylet knob height	3.4	3.2 ± 0.64 (2.7-3.9)	3.3 ± 0.43 (2.9-3.7)	3.1 ± 0.52(2.7-3.5)
Stylet knob width	2.9	2.9 ± 0.36 (2.5-3.2)	2.7 ± 0.1 (2.6-2.8)	2.4 ± 0.23 (2.2-2.6)
Pharynx length	85	109 ± 21.9 (82-132)	88 ± 8.9 (78-99)	95 ± 24.6 (72-121)
Ant. end to median bulb valve	50	69 ± 12.3 (53-78)	63 ± 7.0 (58-72)	71 ± 20.8 (56-86)
Ant. end to post. end of gland	93	128 ± 23.6 (91-148)	116 ± 17.6 (102-142)	122 ± 49 (88-157)
Diam. at mid-body	33	34 ± 5.2 (28.9-43)	32 ± 2.2 (29-34)	40 ± 5.5 (34-44)
Diam. at anus	23.4	24.1 ± 1.3 (22.0-25.2)	23.6 ± 2.1 (22.3-26.8)	27.4 ± 3.7 (23.2-30)
Median bulb length	11.5	14.7 ± 1.3 (13.5-16.5)	14.5 ± 0.0(14.5-14.5)	–
Median bulb diam.	13.5	12.6 ± 1.2(11.0-14.0)	11.5 ± 0.0(11.5-11.5)	–
Median bulb valve length	–	3.6 ± 0.22 (3.5-4.0)	3.0 ± 0.0 (3.0-3.0)	–
Median bulb valve width	–	2.6 ± 0.22 (2.5-3.0)	2.0 ± 0.0 (2.0-2.0)	–
Lipregiondiam.	10.7	9.3 ± 0.43 (8.7-9.7)	8.7 ± 1.1 (7.6-10.1)	11.0 ± 1 (10.3-11.7)
Lip region height	7.4	6.0 ± 0.72 (5.2-6.9)	5.9 ± 1.5 (4.2-7.9)	5.9 ± 0.8 (5.3-6.5)
Tail	16.0	18.4 ± 1.9 (16.5-21.5)	16.8 ± 1.7 (15.0-19.0)	32 ± 3.0 (29.5-35)
Scutellum length	–	5.5 ± 0.36(5.2-6.1)	4.1 ± 0.88 (3.0-5.1)	3.9 ± 0.93 (3.3-4.6)
Scutellum width	–	5.0 ± 0.3 (4.6-5.4)	3.9 ± 0.89 (2.9-4.9)	3.3 ± 0.25 (3.1-3.5)
Spermatheca length	–	–	15.8 ± 0.78 (15.2-16.3)	–
Spermatheca diam.	–	–	15.1 ± 0.82(14.6-15.7)	–
Gonad anterior length	–	56 ± 0.0 (56-56)	52 ± 14.2(36-61)	91 ± 0.0 (91-91)
Gonad posterior length	–	–	59 ± 0.0 (59-59)	–
Spicule length	–	–	–	–
Ant. end to S-E/pharynx length	–	0.98 ± 0.02 (0.97-1.0)	1.1 ± 0.13 (0.89-1.2)	1.1 ± 0.03 (1.1-1.2)

S-E = secretory/excretory pore position.

**Table 5:** Morphometrics of female of Scutellonema clathricaudatum type B from Nigeria. All measurements are in *μ*m and in the form: mean ± s.d. (range).

Character	Sample						
	4NS9-1	4NS11-1	2NS29-1	2NS29-5	2NS29-7	2NS29-13	2NS29-19
n	1	3	2	4	3	2	1
L	710	843 ± 95 (739-925)	804, 792	770 ± 53 (719-840)	784 ± 33 (746-805)	872, 785	797
a	22.5	23.1 ± 2.2 (21.5-25.5)	23.0, 24.1	20.3 ± 1.4(19.1-22.1)	20.7 ± 2.7 (19.0-23.8)	25.1, 20.6	19.3
b	7.2	8.3 ± 0.49 (8.0-8.7)	7.4, 7.6	8.8 ± 0.82 (8.1-10)	6.7 ± 0.53 (6.3-7.3)	7.0, 6.5	9.2
b′	6.0	6.8 ± 0.78 (6.2-7.3)	6.8, 6.8	6.9 ± 0.98 (5.9-8.2)	5.9 ± 0.78 (5.4-6.7)	6.5, 5.7	7.4
c	26.3	40.4 ± 6.8 (35.2-48.1)	26.4, 27.3	29.8 ± 1.9 (27.7-31.6)	33.4 ± 1.6 (31.8-35)	37.1, 29.6	27.9
c′	1.2	0.83 ± 0.16 (0.67-0.99)	1.1, 1.2	1.0 ± 0.1 (0.95-1.2)	0.94 ± 0.06 (0.9-1.0)	1.1, 1.1	1.1
o	–	23.4 ± 4.1 (20.5-26.3)	27.8, 23.7	24.8 ± 4.9 (18.9-30.0)	27.1 ± 4.6 (22.3-31.4)	34.3, 33.8	19.7
V	53.3	53.5 ± 2.1 (51.3-55.5)	52.3, 53.3	51.9 ± 1.1 (50.3-52.7)	55.1 ± 1.8 (53.0-56.5)	54.3, 52.7	54.2
Stylet	26.0	27.3 ± 2.0 (25.5-29.5)	26.0, 26.0	26.3 ± 1.2(25.5-28.0)	25.2 ± 0.76 (24.5-26.0)	26.5, 25.5	26.5
Conus	12.0	12.0 ± 1.0 (11.0-13.0)	11.5, 12.5	12.0 ± 0.5 (11.5-12.5)	11.3 ± 0.29 (11.0-11.5)	12.0, 11.0	12.0
Shaft and knobs	14.0	15.3 ± 1.0 (14.5-16.5)	14.5, 13.5	14.3 ± 1.0 (13.5-15.5)	13.8 ± 0.58 (13.5-14.5)	14.5, 14.5	14.5
Stylet width	2.2	2.2 ± 0.03 (2.2-2.2)	–	2.0 ± 0.14 (1.9-2.2)	2.4 ± 0.36 (2.0-2.8)	2.0, 2.0	2.2
m	46.2	43.9 ± 0.67 (43.1-44.4)	44.2, 48.1	45.6 ± 1.3 (44.6-47.1)	45.0 ± 0.89 (44.2-46.0)	45.3, 43.1	45.3
Stylet knob height	3.0	3.7 ± 0.49 (3.4-4.1)	3.0	3.2 ± 0.07 (3.1-3.3)	3.0 ± 0.24 (2.8-3.3)	3.5, 3.5	2.9
Stylet knob width	3.2	3.0 ± 0.49 (2.7-3.4)	2.3	2.3 ± 0.25 (2.1-2.6)	2.7 ± 0.15 (2.6-2.8)	3.3, 3.3	2.4
Pharynx length	99	108 ± 1.2(107-109)	109, 104	88 ± 2.3 (84-89)	118 ± 9.1 (110-128)	124, 122	87
Ant. end to median bulb valve	61	78 ± 4 (75-80)	67, 79	64 ± 11.9 (56-82)	78 ± 4.7 (73-82)	86, 86	62
Ant. end to post. end of gland	119	132 ± 9 (126-139)	119,117	113 ± 13.7 (103-133)	135 ± 15 (119-148)	135,137	107
Diam. at mid-body	32	37 ± 3.4 (33-40)	35, 33	38 ± 4.4 (32-43)	38 ± 3.8 (34-41)	35, 38	41
Diam. at anus	22.4	25.5 ± 1.2 (24.7-27)	27.6, 23.7	25.2 ± 3.5 (22.2-30.0)	25.0 ± 1.9 (22.8-26.6)	22.2, 25.1	26.2
Median bulb length	13.5	14.3 ± 1.0 (13.5-15.5)	14.0, 15.0	14.3 ± 0.65 (13.5-15.0)	15.7 ± 0.58 (15.0-16.0)	15.0, 14.0	14.0
Median bulb diam.	13.0	11.3 ± 0.29 (11.0-11.5)	13.5, 9.0	11.1 ± 0.95 (10.5-12.5)	11.2 ± 0.29 (11.0-11.5)	11.5,10	10.5
Median bulb valve length	3.5	3.3 ± 0.29 (3.0-3.5)	3.5, 4.0	3.1 ± 0.25 (3.0-3.5)	3.3 ± 0.29 (3.0-3.5)	5.0, 3.0	3.5
Median bulb valve width	2.5	2.5 ± 0.0 (2.5-2.5)	2.5, 3.0	2.5 ± 0.41 (2.0-3.0)	2.3 ± 0.76(1.5-3.0)	3.0, 2.5	2.5
Lip region diam.	9.3	10.6 ± 0.32(10.4-10.9)	10.8, 10.1	10.3 ± 0.36(10.1-10.9)	9.7 ± 0.43 (9.3-10.1)	10.6, 10.7	10.8
Lip region height	4.1	5.8 ± 0.1 (5.7-5.8)	6.5, 6.0	6.1 ± 0.33 (5.8-6.6)	6.2 ± 0.4 (5.9-6.7)	6.7, 6.9	6.3
Tail	27.0	21.2 ± 3.3 (18.0-24.5)	31, 29.0	25.9 ± 2.3 (23.5-29.0)	23.5 ± 0.5 (23.0-24.0)	23.5, 26.5	28.5
Scutellum length	4.0	4.7 ± 0.14 (4.6-4.9)	4.7, 4.0	4.5 ± 0.51 (3.8-4.9)	4.4 ± 0.04 (4.4-4.5)	4.2, 4.2	4.3
Scutellum width	5.1	4.3 ± 0.3 (4.1-4.7)	4.7, 4.2	4.0 ± 0.59 (3.1-4.5)	3.9 ± 0.27 (3.6-4.1)	3.1, 3.9	4.3
Spermatheca length	–	16.7 ± 0.0(16.7-16.7)	–	–	–	–	–
Spermatheca diam.	–	14.4 ± 0.0(14.4-14.4)	–	–	–	–	–
Gonad anterior length	–	82 ± 11 (69-90)	–	–	78 ± 0.0 (78-78)	–	–
Gonad posterior length	34	–	–	–	66 ± 0.0 (66-66)	–	–
Spicule length	–	–	–	–	–	–	–
Ant. end to S-E/pharynx length	0.93	0.96 ± 0.1 (0.89-1.0)	0.91, 1.0	1.0 ± 0.0(1.0-1.0)	0.89 ± 0.02 (0.88-0.91)	0.96, 0.96	1.2

S-E = secretory/excretory pore position.

**Table 6:** Morphometrics of female of *Scutellonema clathricaudatum* type C from Nigeria. All measurements are in *μ*m and in the form: mean ± s.d. (range).

Character	Sample			
	L29	4GS22-1	4GS22-2	2NS7-1
n	2	4	3	9
L	644, 637	838 ± 46 (790-899)	828 ± 19.8 (805-841)	833 ± 90 (683-966)
a	15.8, 15.6	21.8 ± 2.9 (19.2-25.5)	18.1 ± 0.2(17.8-18.2)	23.2 ± 3.3 (18.0-26.1)
b	7.1, 6.5	8.8 ± 3.2 (6.6-13.6)	7.1 ± 0.24 (7.0-7.4)	7.5 ± 0.73 (6.9-9.2)
b′	5.9, 5.7	7.1 ± 1.8 (5.9-9.8)	6.8 ± 0.1 (6.7-6.9)	6.4 ± 1.0 (5.7-8.8)
c	23.4, 24.5	32.5 ± 5.4 (27.7-37.6)	26.5 ± 1.5 (25.4-28.2)	36.5 ± 4.4 (29.1-42.0)
c′	0.99, 0.92	1.1 ± 0.19 (0.82-1.3)	0.98 ± 0.06 (0.94-1.1)	0.88 ± 0.11 (0.76-1.1)
o	19.5	20.4 ± 7.4(12.1-26.4)	23.6 ± 5.7 (17.4-28.5)	27.9 ± 3.5 (24.7-34.9)
V	51.3, 50.8	55 ± 0.59 (54.3-55.7)	50.2 ± 0.11 (50.1-50.2)	55.4 ± 2.1 (51.9-58.7)
Stylet	28, 28	27.6 ± 1.2 (26.0-28.5)	28.3 ± 0.58 (28-29)	26.3 ± 2.3 (21.0-28.5)
Conus	13.5	12.6 ± 0.63 (12.0-13.5)	12.5 ± 0.5 (12.0-13.0)	11.7 ± 1.3 (8.5-13.0)
Shaft and knobs	14.5	15.0 ± 1.5 (13.5-16.5)	15.8 ± 1.0 (15.0-17.0)	14.6 ± 1.2(12.5-16.0)
Stylet width	1.9	2.3 ± 0.28 (2.0-2.7)	2.3 ± 0.32 (2.1-2.7)	2.4 ± 0.28 (2.2-3.0)
m	48.2	45.8 ± 3.3 (42.1-49.1)	44.2 ± 2.6 (41.4-46.4)	44.3 ± 2.1 (40.5-46.4)
Stylet knob height	–	3.4 ± 0.08 (3.4-3.5)	3.9 ± 0.29 (3.6-4.2)	3.6 ± 0.61 (2.3-4.4)
Stylet knob width	–	3.1 ± 0.2 (2.9-3.3)	3.0 ± 0.96 (2.1-4.0)	2.9 ± 0.55 (2.0-3.5)
Pharynx length	91, 99	104 ± 32 (58-126)	116 ± 6.5 (109-120)	112 ± 13.8 (84-127)
Ant. end to median bulb valve	58, 65	67 ± 16.9 (42-78)	73 ± 2.2(71-75)	75 ± 8.3 (59-89)
Ant. end to post end of gland	109, 111	124 ± 29.4 (80-143)	121 ± 1.8 (120-123)	134 ± 11.5 (110-149)
Diam. at mid-body	41, 41	39 ± 6.6 (31-47)	46 ± 0.62 (45-46)	36 ± 5.1 (30.0-44)
Diam. at anus	27.9, 28.4	24.7 ± 4.7 (17.9-28.2)	32 ± 2.6 (30.0-35)	26.3 ± 3.5 (21.3-32)
Median bulb length	14.0	15.8 ± 1.6 (14.0-17.0)	15.8 ± 0.58(15.5-16.5)	15.3 ± 1.4(13.5-18.0)
Median bulb diam.	12.5	12.8 ± 1.6 (11.0-14.0)	12.7 ± 0.76(12.0-13.5)	12.8 ± 1.2(10.5-14.0)
Median bulb valve length	4.5	3.8 ± 0.29 (3.5-4.0)	3.5 ± 0.5 (3.0-4.0)	3.5 ± 0.38 (3.0-4.0)
Median bulb valve width	3.0	2.7 ± 0.58 (2.0-3.0)	2.8 ± 0.29 (2.5-3.0)	2.9 ± 0.42 (2.0-3.5)
Lip region diam.	10.9, 9.9	9.6 ± 0.13 (9.4-9.7)	10.1 ± 0.58 (9.4-10.5)	10.4 ± 0.99 (9.2-11.8)
Lip region height	5.5, 5.4	5.2 ± 0.33 (4.8-5.6)	4.6 ± 0.44(4.1-5)	6.0 ± 1.0 (4.7-7.8)
Tail	27.5, 26.0	26.4 ± 5.2 (21.0-32)	31 ± 2.5 (28.5-33)	23 ± 2.8 (20-29)
Scutellum length	4.2, 5.0	4.6 ± 0.32 (4.3-5.0)	4.9 ± 0.34 (4.6-5.3)	4.5 ± 0.62 (3.6-5.6)
Scutellum width	4.1, 4.6	4.6 ± 0.42 (4.0-4.9)	4.6 ± 0.17 (4.5-4.8)	4.2 ± 0.65 (3.4-5.3)
Spermatheca length	–	19.6 ± 0.0(19.6-19.6)	–	–
Spermatheca diam.	–	15.5 ± 0.0(15.5-15.5)	–	–
Gonad anterior length	–	74 ± 9.6 (67-85)	–	92 ± 0.0 (92-92)
Gonad posterior length	63	–	–	–
Spicule length	–	–	–	–
Ant. end to S-E/pharynx length	–	0.95 ± 0.16(0.85-1.1)	0.92 ± 0.12(0.84-1.1)	1.1 ± 0.13 (0.99-1.3)

S-E = secretory/excretory pore position.

### Description

#### Female

Body arcuate, C-shaped when relaxed, annuli *ca* 2.1 (2-3 *μ*m) wide at mid-body, lateral fields areolated anteriorly and at level of scutellum, often smooth at mid-body. Lip region hemispherical to conical, slightly to flattened ante-riorly, not offset, slightly offset, to well offset by constriction, with seven (6-8) annuli. Basal lip annulus lacking longitudinal striations, stylet well developed with rounded to oval basal knobs posteriorly and with irregular anterior surface. Excretory pore at level of pharyngeal gland lobe, 109 (80-142) *μ*m from anterior end. Hemizonid 0-1 annulus anterior to excretory pore and 1-4 annuli long. Genital tract often not seen in detail. Spermatheca not developed. Intestine slightly overlapping rectum. Epiptygmata usually present, single or double. Scutellum crescent to rounded in shape, located opposite or slightly anterior or posterior to anus. Tail conoid, round to squarish and ventrally curved, 0.94 (0.67-1.3) anal body diam. long and with 16 (11-21) annuli, terminus of variable shape.

### Remarks

The 15 populations showed considerable morphological and molecular variation, which could be assigned into four groups A, B, C and D, based on minor morphological and morphometric differences associated with molecular clades (for at least one of the markers). The four types all fit within *S. clathricaudatum* as defined by Germani *et al*. (1985a): *i*) *S. clathricaudatum* type A (three populations) characterised by having a continuous lip region and tail short, round to squarish (tail = 17.5 (15.0-21.5) *μ*m; c = 43.2 (30.1-50.9); c′ = 0.74 (0.67-0.98)); *ii*) *S. clathricaudatum* type B (seven populations) char-acterised by having an offset lip region and tail conoid and rounded (tail = 25.2 (18.0-31.0) *μ*m; c = 32.0 (26.3-48.1); c′ = 1.0 (0.7-1.2)); *iii*) *S. clathricaudatum* type C (four populations) characterised by having lip region slightly offset to offset and tail slightly tapering with squarish end (tail = 25.6 (20.0-33.0) *μ*m; c = 32.6 (23.4-42.0); c′ = 0.95 (0.76-1.3)); and *iv*) *S. clathricaudatum* type D (one population) characterised by having a broader lip width, lateral field areolated at tail level and its tail length and shape (conoid).

### Diagnosis and Relationships

*Scutellonema clathricaudatum* is similar to *S. conicephalum* with respect to the absence of males and the spermatheca, the lack of longitudinal striae on the basal lip annulus and with areolation at scutellum level. However, *S. clathricaudatum* can be distinguished by having 6-8 lip annuli *vs* three in *S. conicephalum*.

The intraspecific molecular variation in *S. clathricau-datum sensu lato* is high; 0-14 bp (0-2.2%) and 0-52 bp (0-13%) for D2-D3 and *COI*, respectively. The *Scutellonema* sequences form a weakly supported clade according to *COI* and are not resolved according to the D2-D3 analysis.

Although some molecular clades within *S. clathricau-datum* were found associated with some minor morphological differences, species delimitation did not appoint distinct taxonomic identities within *S. clathricaudatum* (no significant Rosenberg’s P_AB_ values).

### Note

In 1959, Whitehead described two new species, *Hoplolaimus aberrans* and *S. clathricaudatum*. For *H*. *aberrans*, the phasmids were referred to as scutella and it is therefore not clear why this species was categorised within *Hoplolaimus* instead of *Scutellonema*, although this was probably based on the lip region morphology.

Sher (1964), revising the genus, transferred *H*. *aberrans* to *Scutellonema* and separated *S. aberrans* and *S. clathricaudatum* based on the lip region morphology (distinctly offset *vs* slightly or not offset). However, Germani *et al*. (1985a) considered *S. aberrans* as a junior synonym of *S. clathricaudatum*, based on the variation in lip shape within individuals in the type populations of *S. clathricau-datum* and *S. aberrans*. In their key to the genus, Germani *et al*. (1985a) proposed *S. clathricaudatum sensu lato* as including all species without males or developed spermatheca, lateral field areolated at scutella level, S-E pore at the level of the pharyngeal gland lobe, lip region with 4-9 annuli, and lacking longitudinal striation on the basal annulus. Given the wide diversity of lip region shapes observed in our populations and in the collection specimens from Ghent University Museum – Zoology Collections, Belgium, and the WaNeCo, we agree, for the time being at least, with the proposal of *S. aberrans* as a junior synonym of *S. clathricaudatum*.

**Fig. 7.**
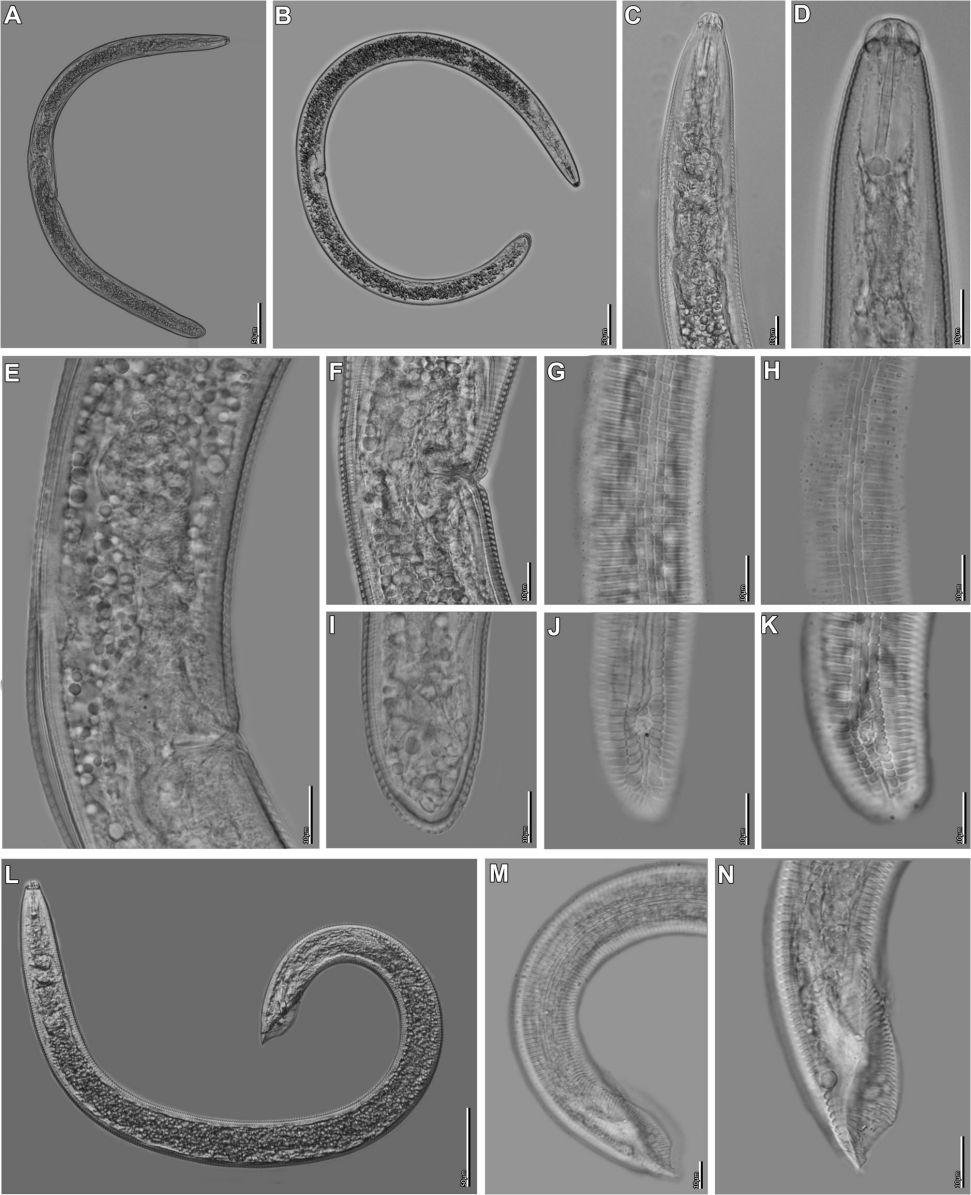
*Scutellonema* sp. D., light micrographs of female (A-K) and male (L-N). A, B: Entire body; C: Pharynx; D: Anterior end; E: Part of reproductive system showing spermatheca; F: Vulval region showing epiptygmata; G, H: Lateral field at mid-body; I: Tail; J, K: Lateral field at scutellum; L: Entire body; M: Posterior region; N: Tail region. (Scale bars: A, B, L = 50 *μ*m; C-K, M, N = 10 *μ*m.)

Four populations, collected from yam rhizosphere in separate locations in Nigeria and Ghana represent *Scutellonema* sp. D, primarily based on molecular data.

### Measurements

See Table 7.

**Table 7:** Morphometrics of female and male of *Scutellonema* sp. D from Ghana and Nigeria and *Scutellonema* sp. 2 from Nigeria. All measurements are in *μ*m and in the form: mean ± s.d. (range).

Character	Sample							
	*Scutellonema* sp. D					*Scutellonema* sp. 2		
	4GS15-1		4GS17-1	2NS30-1		2NS32-1	2NS23-9	2NS23-13	
	Female	Male	Female	Female	Female	Female	Female	Male
n	1	1	3	2	2	4	3	4
L	727	578	730 ± 73 (647-781)	831, 837	631, 725	806 ± 19.6 (789-827)	661 ± 29.1 (637-693)	666 ± 36 (619-705)
a	22.0	19.0	24.0 ± 1.7 (22.0-25.0)	20.0, 24.0	20.0, 20.0	19.1 ± 1.8 (17.5-21.6)	24.0 ± 1.3 (22.5-25.2)	25.5 ± 3.5 (20.4-28.0)
b	7.4	6.4	7.9 ± 0.0 (7.9-7.9)	8.4, 11.0	6.4, 6.1	8.7 ± 0.85 (7.8-9.6)	6.7 ± 0.23 (6.5-6.9)	7.0 ± 0.85 (6.4-8.2)
b′	5.7	5.6	5.8 ± 0.49 (5.4-6.1)	6.0, 7.8	4.6, 5.2	5.9 ± 0.89 (5.3-6.5)	5.1 ± 0.19 (5.0-5.4)	5.5 ± 0.75 (4.8-6.3)
c	42.0	22.0	33.0 ± 3.6 (29.0-36.0)	36.0, 38.0	41.0, 37.0	35.0 ± 8.9 (26.7-44.7)	30.0 ± 4.5 (25.0-33.8)	26.0 ± 2.3 (23.7-28.2)
c′	0.9	1.4	0.9 ± 0.0 (0.9-0.9)	0.9, 0.9	0.7, 0.8	0.88 ± 0.15 (0.74-1.0)	1.1 ± 0.09 (0.99-1.2)	1.4 ± 0.16 (1.2-1.6)
o	28.0	–	24.3 ± 4.9 (21.0-30.0)	31.0	22.0, 23.0	16.6 ± 4.5 (12.7-20.7)	25.8 ± 6.0 (19.5-31.5)	29.1 ± 5.0 (21.9-32.8)
V	57.0	–	58.0 ± 2.6 (56.0-61.0)	57.0, 55.0	58.0, 58.0	56.7 ± 0.57 (56.1-57.4)	59.0 ± 1.6 (58.0-60.9)	–
Stylet	22.5	23.0	24.8 ± 0.58 (24.5-25.5)	26.5, 27.5	26.5, 27.5	25.8 ± 3.0 (23.0-29.0)	23.7 ± 0.58 (23.0-24.0)	23.1 ± 1.2(21.5-24.0)
Conus	9.0	–	11.0 ± 0.0(11.0-11.0)	12.0, 12.0	11.5, 12.5	10.6 ± 1.9 (8.5-12.5)	11.8 ± 1.1 (11.0-12.5)	10.9 ± 1.3 (9.5-12.5)
Shaft and knobs	13.5	–	13.8 ± 0.58(13.5-14.5)	14.5, 15.5	15.0, 15.0	15.1 ± 1.4(13.5-17.0)	11.8 ± 0.35 (11.5-12.0)	12.3 ± 0.87(11.5-13.5)
Stylet width	2.1	–	2.2 ± 0.28 (2.0-2.5)	2.5, 1.8	2.0, 2.2	2.0 ± 0.44(1.7-2.3)	1.9 ± 0.0 (1.9-1.9)	1.7 ± 0.28 (1.4-2.0)
m	40.0	–	44.3 ± 1.2 (43.0-45.0)	45.0, 44.0	43.0, 45.0	41.1 ± 3.8 (36.2-45.5)	50.0 ± 3 (47.8-52.1)	47.0 ± 3.9 (43.8-52.1)
Stylet knob height	–	–	2.7 ± 0.0 (2.7-2.7)	2.9, 3.6	2.8, 3.0	2.6 ± 0.08 (2.6-2.7)	3.3 ± 0.0 (3.3-3.3)	2.7 ± 0.36 (2.3-3.1)
Stylet knob width	–	–	2.7 ± 0.0 (2.7-2.7)	2.7, 5.2	2.4, 2.2	2.6 ± 0.16 (2.4-2.7)	2.0 ± 0.0 (2.0-2.0)	2.0 ± 0.25 (1.8-2.3)
Pharynx length	98	90	99 ± 0.0 (99-99)	99, 76	99, 120	93 ± 7.1 (85-101)	99 ± 3.2 (95-101)	96 ± 11.5 (81-108)
Ant. end to median bulb Valve	70	55	68 ± 5.5 (64-74)	66, 57	76, 84	74 ± 8.4 (65-83)	67 ± 3.2 (64-70)	64 ± 9.0 (54-75)
Ant. end to post. end of gland	128	104	124 ± 6.0 (119-128)	139, 107	136, 139	138 ± 24.3 (121-155)	129 ± 10 (119-139)	123 ± 18.3 (105-147)
Diam. at mid-body	33	31	30 ± 0.65 (29.7-31)	43, 34	32, 36	42 ± 3.2 (38-45)	27.6 ± 1.4 (26.2-29.0)	26.5 ± 4.1 (23.7-33)
Diam. at anus	19.0	18.6	24.3 ± 0.35 (24.1-24.7)	27.0, 24.2	21.0, 24.2	27.1 ± 2.2 (24.4-29.0)	20.5 ± 1.3 (19.0-21.7)	18.5 ± 3.2(16.2-23.3)
Median bulb length	15.0	–	13.3 ± 2.3 (12.0-16.0)	13.5, 16.5	14.0, 17.0	13.1 ± 2.2(11.0-15.0)	14.7 ± 0.76(14.0-15.5)	13.0 ± 1.7 (11.0-15.0)
Median bulb diam.	10.0	–	10.3 ± 1.6 (8.5-11.5)	15.0, 12.0	10.5, 12.0	14.6 ± 1.8 (13.0-17.0)	12.0 ± 1.0 (11.0-13.0)	10.5 ± 1.0 (10.0-12.0)
Median bulb valve length	6.5	–	3.3 ± 0.29 (3.0-3.5)	3.5, 3.5	3.5, 3.0	3.3 ± 0.29 (3.0-3.5)	3.5 ± 0.87 (3.0-4.5)	3.1 ± 0.25 (3-3.5)
Median bulb valve width	2.0	–	2.5 ± 0.0 (2.5-2.5)	3.0, 2.5	2.5, 2.5	2.6 ± 0.48 (2.0-3.0)	2.5 ± 0.0 (2.5-2.5)	2.1 ± 0.25 (2.0-2.5)
Lipregiondiam.	8.7	8.6	9.7 ± 0.98 (8.7-10.7)	10.8, 10.3	10.2, 10.5	10.7 ± 0.3 (10.3-11.0)	10.1 ± 0.49 (9.7-10.6)	9.3 ± 0.33 (8.9-9.7)
Lip region height	4.5	3.7	4.6 ± 0.46 (4.2-5.1)	6.2, 5.0	6.6, 6.9	6.0 ± 0.17(5.8-6.2)	5.8 ± 1.4 (4.6-7.4)	5.1 ± 0.29 (4.7-5.4)
Tail	17.5	26.0	22.2 ± 1.0 (21.0-23.0)	23.0, 22.0	15.5, 19.5	24.1 ± 5.9(18.5-29.5)	22.3 ± 2.8 (20.5-25.5)	25.8 ± 1.7 (24-28)
Scutellum length	4.0	3.9	4.4 ± 0.28 (4.1-4.6)	4.6, 5.3	4.8, 4.8	3.3 ± 0.24 (3.1-3.5)	3.7 ± 0.61 (3.0-4.2)	3.8 ± 0.18 (3.6-4.0)
Scutellum width	3.6	3.4	4.4 ± 0.37 (4.1-4.8)	4.7	4.8, 4.6	2.8 ± 0.19(2.6-2.9)	3.6 ± 0.12(3.5-3.7)	3.7 ± 0.24 (3.5-4.1)
Spermatheca length	19.4	–	18.5 ± 1.9 (17.1-19.9)	–	12.6, 14.0	–	–	–
Spermatheca diam.	19.0	–	10.7 ± 0.0(10.7-10.7)	–	11.3, 13.9	–	–	–
Gonad anterior length	–	–	68 ± 9.1 (59-78)	–	66, 80	–	–	252 ± 0.0 (252-252)
Gonad posterior length	67	–	–	–	–	–	–	–
Spicule length	–	31	–	–	–	–	–	29.8 ± 1.4 (27.8-31)
Ant. end to S-E/pharynx length	1.1	0.72	–	–	1.0, 0.98	1.0 ± 0.0 (1.0-1.0)	1.1 ± 0.04(1.0-1.1)	1.0 ± 0.07 (0.96-1.1)

S-E = secretory/excretory pore position.

### Description

#### Female

Body slightly ventrally curved to spiral. Cuticle at mid-body with 1.8 *μ*m annuli wide. Lateral fields one-sixth diam. of mid-body, completely areolated at anterior portion of body and at tail level (from anterior region of scutella to tail end) and partially to completely areolated at mid-body. Lip region broadly rounded, slightly flattened anteriorly and slightly offset from body, with 6-7 annuli. Basal lip annulus without longitudinal striations. Stylet well developed. Stylet knobs, rounded posteriorly, flattened and slightly indented anteriorly, 3.0 (2.7-3.6) *μ*m wide and 3.0 (2.2-5.2) *μ*m high. Conus shorter than shaft and knobs, m = 43.8 (40-45)%. Median bulb spherical to oblong. Pharyngeal gland lobe overlapping intestine dorsally. Excretory pore situated at nerve ring level, 107 (99-117) *μ*m from anterior end. Hemizonid two annuli long, situated opposite excretory pore. Spermatheca thick-walled and either empty or filled with sperm cells. Vagina with not well developed ‘vaginal glands’. Epiptygmata single to double, not observed in some cases (double in original description). Intestine not overlapping rectum. Scutellum moderate to large size, crescent to rounded in shape, situated opposite anus to posterior to anus. Tail straight to ventrally curved, 0.86 (0.7-0.9) anal body diam. long and with 15 (13-19) annuli.

#### Male

Similar to female except for reproductive structures with a broadly enveloping bursa.

### Diagnosis and Relationships

*Scutellonema* sp. D is similar to *S. clathricaudatum* and *S. cavenessi*. It is distinguished from *S. clathricaudatum* by the presence of the spermatheca and males and from *S. cavenessi* by the areolation of the lateral field at mid-body (partially areolated *vs* partially striated) and spermatheca obscure and reduced in *Scutellonema* sp. D *vs* generally developed and filled with sperm cells in *S. cavenessi*.

*Scutellonema* sp. D sequences form a well supported clade (PP = 0.95; PP = 0.96) with an intraspecific variation of 0-7 bp (0-1%) and 1-19 bp (0.3-5.7%) (Figs 3, 4) based on the D2-D3 and *COI* tree topologies, respectively. The interspecific divergence between *Scutellonema* sp. D and *S. cavenessi* were 16-24 bp (2.4-3.5%) and 51-60 bp (14.4-17%); and between *Scutellonema* sp. D and *S. clathricaudatum sensu lato*, 6-14 bp (0.8-2.2%) and 38-47 bp (9.7-14%) based on D2-D3 and *COI*, respectively. Taxonomic distinctness of *Scutellonema* sp. D was also supported by a significant Rosenberg’s P_AB_ of 1.8E−6 (Fig. 3) and 3.7E−7 (Fig. 4) based on D2-D3 and *COI*, respectively.

### Remarks

*Scutellonema* sp. D populations are morphologically similar to the description provided by Van den Berg *et al*. (2013), with 0-6 bp (0-0.9%) D2-D3 and 1-17 bp (0.3-4.4%) *COI* sequences difference and they cluster with maximal support in the same clade as the population reported by Van den Berg *et al*. (2013).

**Fig. 8.**
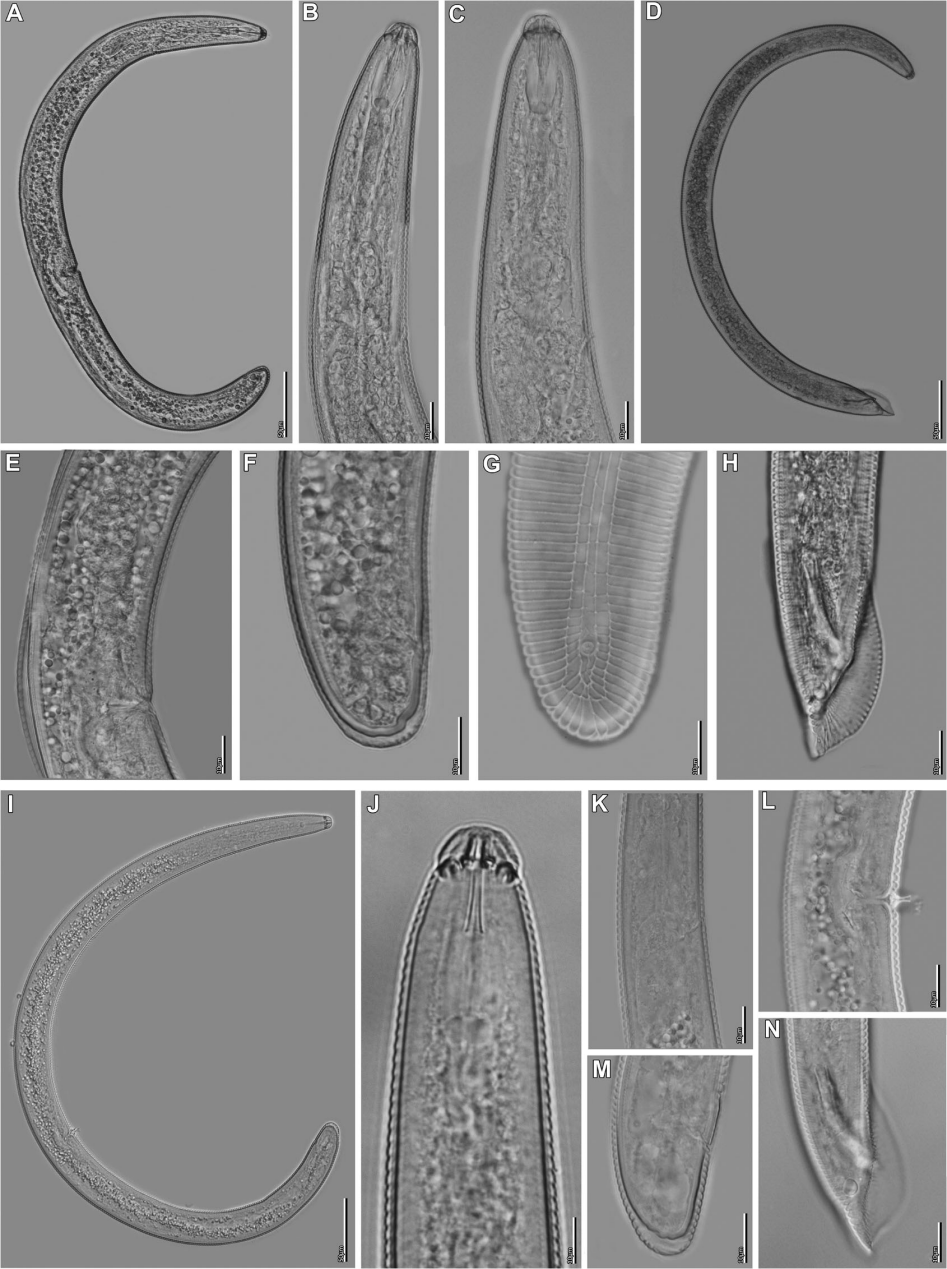
*Scutellonema* sp. 1 (A-H), light micrographs of female (A-C, E-G) and male (D, H). A: Entire body; B, C: Pharynx; D: Entire body male; E: Part of reproductive system showing functional spermatheca; F: Tail; G: Lateral field at scutellum; H: Male tail. *Scutellonema* sp. 2 (I-N), light micrographs of female (I-M) and male (N); I: Entire body; J: Anterior end; K: Part of pharynx showing S-E pore; L: Epiptygmata; M: Tail; N: Male tail. (Scale bars: A, D, I = 50 *μ*m; B, C, E-H, J-N = 10 *μ*m.)

Four populations, collected from yam rhizosphere from four locations in Ghana represent *Scutellonema* sp. 1.

### Measurements

See Table 8.

**Table 8:** Morphometrics of female and male of *Scutellonema* sp. 1 from Ghana. All measurements are in *μ*m and in the form: mean ± s.d. (range).

Character	Sample					
	L31	4GS27-1		4GS28-1		4GS12-1
	Female	Female	Male	Female	Male	Male
n	2	3	4	13	3	1
L	801, 632	618 ± 91 (534-715)	620 ± 49 (575-683)	724 ± 63 (658-886)	617 ± 29.7 (583-635)	533
a	18.6, 21.4	17.0 ± 1.2(15.9-18.2)	19.5 ± 0.82(18.7-20.3)	21.1 ± 3.9(15.8-29.8)	18.7 ± 2.7 (15.7-20.9)	15.7
b	11.1, 7.3	6.7 ± 1.4 (5.1-7.6)	5.5 ± 1.1 (4.8-6.7)	7.0 ± 0.98 (5.5-9.4)	6.5 ± 0.42 (6.0-6.8)	5.7
b′	8.2, 7.2	5.2 ± 0.86 (4.2-5.7)	5.0 ± 0.84 (4.4-5.9)	5.9 ± 0.61 (4.8-7.1)	4.9 ± 0.15 (4.8-5.1)	4.7
c	32, 34.1	33.7 ± 4.9 (28.9-38.7)	24.7 ± 1.6 (23.1-27.0)	33.8 ± 5.4 (22.0-44)	25.0 ± 0.59 (24.4-25.4)	24.2
c′	0.8, 0.84	0.79 ± 0.06 (0.74-0.85)	1.3 ± 0.22(1.0-1.5)	0.9 ± 0.21 (0.59-1.3)	1.1 ± 0.39 (0.68-1.4)	1.2
o	14.3	31 ± 0.92 (30.3-31.6)	33.6 ± 0.0 (33.6-33.6)	24.1 ± 5.4(17.7-32.7)	27.5 ± 6.9 (23.5-35.4)	–
V	55.1, 58.4	57.2 ± 1.6 (55.8-59.0)	–	57.2 ± 2.6 (52.8-62.0)	–	–
Stylet	29.5, 23.5	23.8 ± 0.76 (23-24.5)	22.5 ± 0.71 (21.5-23.0)	25.0 ± 1.3 (23.0-27.0)	23.8 ± 0.29 (23.5-24.0)	22.5
Conus	13.5, 10.5	10.8 ± 0.76 (10-11.5)	11.0 ± 0.41 (10.5-11.5)	11.5 ± 1.1 (9.5-13.0)	11.8 ± 0.29(11.5-12.0)	10.5
Shaft and knobs	16.0, 13.0	13.0 ± 1.3 (12.0-14.5)	11.5 ± 1.1 (10.0-12.5)	13.5 ± 1.2(12.5-17.0)	12.0 ± 0.0 (12.0-12.0)	12.0
Stylet width	2.0, 1.9	2.0 ± 0.02 (2.0-2.0)	1.8 ± 0.01 (1.8-1.8)	2.1 ± 0.34(1.7-2.7)	1.9 ± 0.12(1.8-2.0)	–
m	45.8, 44.7	45.5 ± 4.1 (40.8-47.9)	49.0 ± 3.3 (45.7-53.5)	45.9 ± 3.5 (35.8-50)	49.6 ± 0.61 (48.9-50.0)	46.7
Stylet knob height	3.7, 2.8	2.9 ± 0.12(2.8-3.0)	2.7 ± 0.67 (2.2-3.4)	3.2 ± 0.5 (2.6-4.1)	2.5 ± 0.29 (2.2-2.7)	–
Stylet knob width	2.7, 1.9	1.8 ± 0.18(1.7-1.9)	2.0 ± 0.46(1.6-2.5)	2.5 ± 0.53 (1.7-3.2)	2.4 ± 0.17 (2.2-2.5)	–
Pharynx length	72, 86	94 ± 11.4 (82-105)	111 ± 15.1 (94-123)	105 ± 9.2 (89-119)	95 ± 1.8 (93-97)	94
Ant. end to median bulb valve	54, 66	65 ± 5.3 (59-70)	66 ± 9.3 (57-75)	68 ± 3.9 (64-78)	65 ± 1.9 (62-66)	60
Ant. end to post. end of gland	97, 88	121 ± 11.6 (107-128)	122 ± 13.3 (107-131)	124 ± 8.7 (116-146)	123 ± 3.8 (120-126)	113
Diam. at mid-body	43, 29	36 ± 2.9 (33-39)	32 ± 3.7 (29-36)	35 ± 5.7 (28-44)	33 ± 3.5 (30-37)	34
Diam. at anus	31.0, 21.9	23.3 ± 1.8 (21.7-25.2)	19.4 ± 3.3(15.3-23.0)	24.8 ± 3.8 (18.2-32)	23.7 ± 8.6 (18.7-34)	18
Median bulb length	14.0, 11.5	13.7 ± 0.58 (13.0-14.0)	13.0 ± 0.5 (12.5-13.5)	15.7 ± 1.8(12.5-18.5)	11.7 ± 1.6 (10.5-13.5)	–
Median bulb diam.	12.5, 9.5	11.5 ± 1.0 (10.5-12.5)	10.7 ± 0.29 (10.5-11.0)	11.5 ± 1.0(10.0-13.5)	11.0 ± 1.8 (9.5-13.0)	–
Median bulb valve length	4.0	3.7 ± 0.58 (3.0-4.0)	3.0 ± 0.0 (3.0-3.0)	3.4 ± 0.4 (3.0-4.0)	2.7 ± 0.29 (2.5-3.0)	–
Median bulb valve width	2.5	2.7 ± 0.29 (2.5-3.0)	2.2 ± 0.29 (2.0-2.5)	2.6 ± 0.36 (2.0-3.0)	2.2 ± 0.76 (1.5-3.0)	–
Lip region diam.	10.1, 9.4	9.0 ± 0.79 (8.1-9.6)	9.1 ± 0.37 (8.8-9.6)	9.7 ± 0.81 (8.5-11.2)	8.4 ± 0.82 (7.9-9.4)	9.2
Lip region height	5.4, 4.8	4.3 ± 0.54 (3.9-4.9)	4.5 ± 0.62 (3.8-5.3)	5.2 ± 0.82 (4.2-7.5)	4.4 ± 0.23 (4.2-4.6)	4.8
Tail	25.0, 18.5	18.3 ± 0.29 (18.0-18.5)	25.1 ± 2.1 (23.5-28.0)	21.9 ± 3.6(15.0-30.0)	24.7 ± 1.5 (23.0-26.0)	22.0
Scutellum length	5.0	4.2 ± 0.32 (4.0-4.6)	3.6 ± 0.65 (2.8-4.0)	4.7 ± 0.51 (3.7-5.6)	4.2 ± 1.5 (3.4-6.0)	3.3
Scutellum width	5.4	4.3 ± 0.66 (3.7-5.0)	2.7 ± 0.15 (2.6-2.9)	4.5 ± 0.39 (3.8-5.2)	4.0 ± 1.5 (3.1-5.7)	3.4
Spermatheca length	–	13.2 ± 3.4(10.8-15.5)	–	15.4 ± 2.0 (13.0-17.4)	–	–
Spermatheca diam.	–	13.6 ± 4.0(10.8-16.4)	–	10.7 ± 1.1 (8.9-12.1)	–	–
Gonad anterior length	–	76 ± 24.9 (58-93)	131 ± 0.0 (131-131)	74 ± 17.9 (54-101)	272 ± 0.0 (272-272)	–
Gonad posterior length	–	75 ± 6.5 (70-80)	–	53 ± 19.7 (30-65)	–	–
Spicule length	–	–	28.9 ± 4.4 (23.7-34)	–	28.9 ± 2.3 (27-31)	27.1
Ant. end to S-E/pharynx length	–	0.87 ± 0.06 (0.82-0.91)	0.68 ± 0.0 (0.68-0.68)	0.92 ± 0.08 (0.8-1.0)	0.92 ± 0.0 (0.92-0.92)	0.74

S-E = secretory/excretory pore position.

### Description

#### Female

Body arcuate, C-shaped when relaxed, annuli *ca* 2.1 *μ*m wide at mid-body, lateral fields areolated anteriorly and at level of scutellum, in some cases areolated in additional places. Lip region hemispherical, slightly flattened anteri-orly, usually slightly offset, occasionally well offset, with seven (6-8) annuli. Basal lip annulus without longitudinal striations (using SEM). Stylet well developed with rounded to oval basal knobs posteriorly and with an irregular anterior surface. Excretory pore at level of pharyngeal gland lobe, 96 (85-112) *μ*m from anterior end. Hemizonid 0-2 annuli anterior to excretory pore. Spermatheca not well developed, spherical and small when visible. Vagina often with obscure ‘vaginal glands’, epiptygmata often present and single, double or not observed in rare cases. Tail rounded to gradually tapering towards tail tip, 0.87 anal body diam. long with 10-17 annuli, terminus variably shaped.

#### Male

Similar to female except for reproductive structures. Bursa narrow and not lobe-shaped with abrupt narrowing.

### Diagnosis and Relationships

*Scutellonema* sp. 1 is similar to *S. cavenessi, Scutellonema* sp. D, and *Scutellonema* sp. 2 with respect to the presence of males and absence of longitudinal striae on the basal lip annulus. *Scutellonema* sp. 1 differs from *S. cavenessi* in having the spermatheca relatively smaller *vs* well developed, tail less broadly rounded *vs* rounded, and the S-E ratio (anterior end to S-E/pharynx length = 0.9 (0.8-1.1) in *Scutellonema* sp. 1 *vs* 1.1 (1.0-1.3) in *S. cavenessi*). *Scutellonema* sp. 1 differs from *Scutellonema* sp. 2 in having non-projecting epiptygmata *vs* projecting in *Scutellonema* sp. 2, and with the hemizonid observed at pharyngeal gland level *vs* hemizonid at pharyngo-intestinal junction and nerve ring level).

*Scutellonema* sp. 1 sequences form a well-supported clade (PP = 1.00) with an intraspecific variation 5-8 bp (0.6-0.9%) (Fig. 3) and 2-10 bp (0.5-3.1%) (Fig. 4) based on the D2-D3 and *COI* tree topologies, respectively. Molecular divergences between *Scutellonema* sp. 1 and *S. cavenessi* were 15-20 bp (1.9-3.3%) and 49-70 bp (16.4-19.6%); between *Scutellonema* sp. 1 and *Scutellonema* sp. 2 13-20 bp (1.6-2.1%) and 58-82 bp (17.7-20.9%) from the D2-D3 and *COI*, respectively. Species delimitation support the distinctness of *Scutellonema* sp. 1 (Significant Rosenberg’s P_AB_: 1.1E−8) based on the *COI* tree topology (Fig. 4). However, no significant Rosenberg’s P_AB_ value was observed based on the D2-D3 tree topology, just a single D2-D3 sequence of its sister taxon (*Scutellonema* sp. 2) was available.

Two populations of this species were collected from yam rhizosphere from two locations in Nigeria.

### Measurements

See Table 7.

### Description

#### Female

Body arcuate, C-shaped to spiral when relaxed, annuli *ca* 1.4 *μ*m wide at mid-body, lateral fields areolated ante-riorly and at level of scutellum, in some cases areolated in additional regions. Lip region hemispherical, slightly flattened anteriorly, usually slightly offset, occasionally well offset, with seven (6-7) annuli. Basal lip annulus without longitudinal striations (SEM), stylet well developed with rounded to oval basal knobs and an irregular anterior surface. Excretory pore at level of pharyngeal gland lobe, 103 (98-105) *μ*m from anterior end. Hemizonid one annulus long and situated from directly anterior to two annuli anterior to excretory pore. Spermatheca not developed to spherical and of small size when visible. Vagina often with obscure ‘vaginal glands’, projecting epiptygmata often present, single, double or not observed in some rare cases. Tail rounded to gradually tapering towards tail tip, 0.97 (0.74-1.2) anal body diam. long with 15 (12-20) annuli, terminus variably shaped.

#### Male

Similar to female except for reproductive structures. Bursa lobe-shaped with abrupt narrowing.

### Diagnosis and Relationships

*Scutellonema* sp. 2 is similar to *S. cavenessi, Scutellonema* sp. D, and *Scutellonema* sp. 1 with respect to the presence of males and absence of longitudinal striae on the basal lip annulus. However, *Scutellonema* sp. 2 is distinct from the others by a longer and tapering tail, c′ = 0.97 (0.74-1.20) (*vs* shorter and rounded tail: c′ = 0.75 (0.52-0.95) in *S. cavenessi*; c′ = 0.86 (0.70-0.90) in *Scutellonema* sp. D; c′ = 0.88 (0.59-1.30) in *Scutellonema* sp. 1), thin and longer epiptygmata (*vs* thicker and shorter in *S. cavenessi*, short to absent in *Scutellonema* sp. D and *Scutellonema* sp. 1). *Scutellonema* sp. 2 is also distinguished from *Scutellonema* sp. D and *Scutellonema* sp. 1 by a larger median bulb with 13.5 (11.0-17.0) *μ*m in *Scutellonema* sp. 2 *vs* 11.5 (9.5-13.5) *μ*m in *Scutellonema* sp. 1 and 11.3 (8.5-15.0) *μ*m in *Scutellonema* sp. D. *Scutellonema* sp. 2 is readily distinguished from *Scutellonema* sp. D and *Scutellonema* sp. 1 by the hemizonid located at the posterior level of the pharyngeal gland lobe compared with at the anterior level of the pharyngeal gland lobe.

*Scutellonema* sp. 2 sequences formed a well-supported clade (PP = 1.00) with an intraspecific variation of 13-19 bp (3.2-4.7%) based on the *COI* tree topologies (Fig. 4).

### Other Scutellonema Species Analysed

*Scutellonema brachyurus* was collected from banana rhizosphere, *S. cavenessi* from onion rhizosphere, and *S. paralabiatum* from banana, maize and onion rhizo-sphere. Morphological and morphometric observations (see Beriso, 2014; Nyiragatare, 2014) agreed with the original descriptions.

### Molecular Phylogeny of Scutellonema

The tree topologies based on D2-D3 and *COI* are largely similar and do not show inconsistencies, except for the positions of clades, which are not well supported according to the *COI*-based tree topology (see below). However, the *COI*-based tree topology was better resolved (Figs 3, 4). The D2-D3 of 28S rDNA gene sequence alignment was 677 bp long and contained 73 *Scutellonema* sequences and three outgroup taxa. The *COI* gene sequence alignment was 390 bp in length and contained 82 sequences of *Scutellonema* and two out-group taxa. Intra- and interspecific variation are given at the species description section and on the respective trees (Figs 3, 4).

The Bayesian inference (BI) trees comprised three major, well-supported clades. Clade I, sister to Clade II and III, which included *S. brachyurus* type A, *S. brachyurus* type B, *S. clavicaudatum* Van den Berg, Tiedt, Stanley, Inserra & Subbotin, 2017 (not in *COI*-based tree), *S. paralabiatum, S. truncatum, Scutellonema* sp. A, and *Scutellonema* sp. B; Clade II consisted entirely of *S. bradys*; and Clade III comprised *S. cavenessi, S. clathricaudatum sensu lato, Scutellonema* sp. 1, *Scutellonema* sp. 2 and *Scutellonema* sp. D. In Clade III, some minor differences were observed between the D2-D3 and *COI* analysis. Based on D2-D3, *Scutellonema* sp. 2 was sister to

*Scutellonema* sp. 1 with maximal support, while based on *COI, Scutellonema* sp. 2 was sister to all other *Scutellonema* species in Clade III and *Scutellonema* sp. 1 was sister to *S. cavenessi, Scutellonema* sp. D and *S. clathricaudatum sensu lato*. However, the *COI*-based relation for *Scutellonema* sp. 1 was only weakly supported and therefore the positions of *Scutellonema* sp. 1 and *Scutellonema* sp. 2 should be considered as unresolved.

## Discussion

In 1964, six of the 11 species revised and described by Sher were recorded from Nigeria alone, with over 60% of all valid species of *Scutellonema* reported from Africa (Siddiqi, 2000), demonstrating the high diversity of the genus on the continent. Based on morphology and morphometrics, we identified four morphospecies of *Scutellonema* from yam tubers and yam rhizosphere in Ghana and in Nigeria: *S. bradys, S. cavenessi, S. clathricaudatum*, and *Scutellonema* sp. D. However, phylogenetic analysis based on *COI* and D2-D3 sequences, in combination with a molecular species delimitation method, revealed two additional unknown species, namely *Scutellonema* sp. 1 and *Scutellonema* sp. 2. This indicates a much wider diversity of the genus *Scutellonema* than previously recognised, confirming the need for more robust and accurate diagnostics of the genus.

The species found in the present study are mainly char-acterised by their large number of lip region annuli (*>*5), the presence of areolation at scutella level, the absence of longitudinal striations on the basal lip annulus, and their relatively small stylets (rarely exceeding 30 *μ*m). They all belong to either the amphimictic or partheno-genetic group. The parthenogenetic populations were all categorised within *S. clathricaudatum sensu lato*, which is known for showing large variability in size and tail shape, and with the lip region “rounded and distinctly offset, to truncate and slightly offset or truncate and distinctly offset” (Sher, 1964; Ali *et al.*, 1973; Germani *et al.*, 1985a; Baujard & Martiny, 1995). Based on a combination of morphological and molecular data, four putative clusters (types A, B, C and D) could be observed which all fit morphologically within *S. clathricaudatum*. However, molecular species delimitation could not confirm the taxonomic distinctness of these lineages. The topologies based on both rDNA and mt*COI* are in agreement with Van den Berg *et al*. (2013, 2017), especially in respect to the three major clades (I, II and III) displayed.

However, while Clade III was not well resolved based on rDNA topology, some relationships that were not well supported based on D2-D3 received close to maximal support based on *COI* (*viz*., *Scutellonema* sp. 1 with *Scutellonema* sp. 2; *S. clathricaudatum* type A with *S. clathricaudatum* type D). Nevertheless, all taxa could be identified independently of the used marker. Hence, our results confirm that both the D2-D3 expansion segments and *COI* are useful markers for *Scutellonema* species delimitation. However, the uni-parental inheritance and the high mutation rate in the mitochondrial sequences provide a better differentiation of closely related species (Janssen *et al.*, 2016). This is especially important for the identification and description of hybrid or cryptic species (Powers, 2004; Kanzaki & Giblin-Davis, 2012; Palomares-Rius *et al.*, 2014). A better phylogenetic resolution of *COI* is already well known, based on *Hoplolaimus* (Holguin *et al.*, 2015), *Rotylenchus* (Cantalapiedra-Navarrete *et al.*, 2013) and Rhabditidae (Fonseca *et al.*, 2008), for example. Furthermore, the higher PCR success rate for *COI* compared with D2-D3 experienced here (70 *vs* 40%) identifies *COI* as a preferred and superior marker for *Scutellonema*.

The current study revealed a wide diversity of *Scutellonema* species occurring in the yam rhizosphere in Ghana and Nigeria, although only *S. bradys* was recovered from yam tuber tissue, a finding which is of clear biological interest. Comparing parasitism genes of *S. bradys* and congeners could provide insights into the evolution of endoparasitism in *Scutellonema* and improve our understanding of the molecular basis of host-parasite interactions and endoparasitism in *Scutellonema*. This could be tackled by comparing the transcriptome analyses of *S. bradys* and well selected species from both Clade I and Clade III.

As the only species that appears able to enter and damage tubers, the need for a precise identification of *S. bradys* among its diverse congeners is clear in order to select appropriate management strategies against the yam nematode and to enable accurate monitoring of its distribution aimed at preventing its spread. Extracting nematodes from clean yam peels, without adhering soil, however, could be advised in order to detect only *S. bradys* and rule out other *Scutellonema* spp. not causing damage on yam. However, while *S. cavenessi* and *S. clathricau-datum* occur on most crops across West Africa (Caveness, 1967; Baujard & Martiny, 1995), they can cause signifi-cant damage to groundnut (*Arachis hypogaea* L.) production (Germani *et al.*, 1985b; Sharma *et al.*, 1992). Consequently, the ability to readily and accurately differentiate these three closely related species would be very useful.

Using only morphological-based identification, the margin of error for misidentifying as *S. bradys* the cohabiting *Scutellonema* species from the rhizosphere of yam is high. However, by combining molecular and morphological data, *S. bradys* appears to be a well defined monophyletic group with its morphological and morpho-metric characters aligning clearly with the available data (Sher, 1964; Van den Berg, 1973; Germani *et al.*, 1985a; Humphreys-Pereira *et al.*, 2014).

To facilitate the morphological identification of *Scutellonema* a dichotomous key is proposed. This key is based on the new information of current study and species descriptions by Germani *et al*. (1985a), Siddiqi (2000), and those described since 2000 (*S. bamboosae* Saha, Lal, Singh, Kaushal & Sharma, 2000; *S. himachalensis* Saha, Lal, Singh, Kaushal & Sharma, 2000; *S. coffeae* Giribabu & Saha, 2002; *S. clavicaudatum*). However, compared with Siddiqi (2000), *S. mabelei* Van den Berg & De Waele, 1990 was not included as this species has a pore-like amphid and was originally described as *Rotylenchus mabelei* Van den Berg & De Waele, 1990. It was listed as a species of *Scutellonema* by Siddiqi (2000), although not designated as a new combination, and was therefore probably a *lapsus*. *Scutellonema southeyi* Williams, 1986 and *S. hoabinhiensis* Nguyen & Nguyen, 1993 were not included in Siddiqi (2000) but are added in the current key.

### Key to species of *Scutellonema* (based on Germani *et al.*, 1985a)

1. Spermatheca functional; male present … … ….. 2
  Spermatheca not functional; male absent or rare … ……………………………………….24
2. Lateral field without areolation at level of scutellum ……………………………………….3
  Lateral field with areolation at level of scutellum . . 6
3. Lip annuli absent … … … … … . *S. clavicaudatum*
  Lip annuli present… … … … … … … … … …4
4. Basal lip annulus without longitudinal striae … … ………………………………. *S. africanum*
  Basal lip annulus with longitudinal striae … … … 5
5. Stylet *<* 30 *μ*m; epiptygmata *>* 5 *μ*m.. *S. labiatum*
  Stylet *>* 30 *μ*m; epiptygmata *<* 5 *μ*m ………..……………………………. *S. tsitsikamense*
6. Basal lip annulus without longitudinal striae … … 7
  Basal lip annulus with longitudinal striae … … . . 17
7. Stylet *<* 30 *μ*m…………………………..8
  Stylet *>* 30 *μ*m………………………….16
8. Scutellum well anterior to anal level … … … … ……………………………… *S. propeltatum*
  Scutellum at or posterior to the anal level … … … 9
9. Lip annuli *<* 5…………………………..10
  Lip annuli *>* 5…………………………..11
10. m *>* 50%; bursa without notch … … … *S. minutum*
  m *<* 50%; bursa with notch … … … *S. bamboosae*
11. Lip region not offset ………………. *S. sibrium*
  Lip region offset…………………………12
12. Lip region truncate; pharyngeal lobe short and cap-like……………………….. *S. transvaalense*
  Lip region hemispherical; pharyngeal lobe long and not cap-like … … … … … … … … … … … . 13
13. Spermatheca well-developed … … … … … … . 14
  Spermatheca indistinct … … … … … … … … 15
14. Vaginal glands well sclerotised; spermatheca filled with sperm cells; males abundant… … …*S. bradys*
  Vaginal glands not sclerotised; spermatheca filled with sperm cells; epiptygmata protruding; tail short and rounded; males abundant … … … . *S. cavenessi*
  Spermatheca obscure; epiptygmata not protruding; males not abundant … … … … *Scutellonema* sp. D
15. Epiptygmata protruding; tail conoid; hemizonid at the pharyngeal gland lobe level; scutellum 3-4.5 *μ*m ………………………… *Scutellonema* sp. 2 Epiptygmata not protruding; tail rounded; hemizonid at level of the pharyngo-intestinal junction and the nerve ring level; scutellum 4-6 *μ*m………………………………………. *Scutellonema* sp. 1
16. S.E pore opposite to nerve ring and to hemizonid … …………………………………*S. grande*
  S.E pore more posterior to nerve ring; hemizonid anterior to S-E …………………… *S. validum*
17. Basal lip annulus with faint longitudinal striae …18
  Basal lip annulus with well demarcated longitudinal striae………………………………….19
18. Lip annuli *<* 5; tail rounded; bursa cover only 75% of tail …………………………… *S. dreyeri*
  Lip annuli *>* 5; tail conically pointed; bursa cover the tail …………………… *S. nigermontanum*
19. Longitudinal striae on the basal lip *<* 5; stylet *>* 35 *μ*m …………………………. *S. southeyi*
  Longitudinal striae on the basal lip *>* 5; stylet *<* 35 *μ*m…………………………………20
20. Basal lip annulus with 6 longitudinal striae … …
  21 Basal lip annulus with *>*10 longitudinal striae … 22
21. Lip not set off; female tail with irregular tail shape ………………………………. *S. siamense*
  Lip offset; female tail with regular tail shape … … ………………………………… *S. erectum*
22. Lip region continuous … … … … … … . . *S. cheni*
  Lip region offset…………………………23
23. Longitudinal striae at the basal lip annulus *>* 15; lip annuli 3-4 … … … … … … … … … . . *S. bizanae*
  Longitudinal striae at the basal lip annulus *<* 15; lip annuli 4 … … … … … … … … … . . *S. clariceps*
24. Vaginal wall with dentate formation, tooth-like structure……………………….. *S. dentivaginum*
  Vaginal wall without dentate formation … … … 25
25. Lateral field without areolation at level of scutellum ………………………………………26
  Lateral field with areolation at level of scutellum … ………………………………………35
26. Basal lip annulus without longitudinal striae … . .
  27 Basal lip annulus with longitudinal striae … … . . 31
27. Scutellum *<* 2 *μ*m……………………….28
  Scutellum *>* 2 *μ*m……………………….29
28. Scutellum situated anterior to anus; tail terminus not indented … … … … … … … … … *S. laeviflexum*
  Scutellum situated posterior to anus; tail terminus indented … … … … … … … . . *S. incisicaudatum*
29. Scutellum situated anterior to anus … … … … … ……………………………. *S. paralabiatum*
  Scutellum situated posterior to anus … … … … 30
30. Lip annuli 5; body C-shaped … … *S. himachalensis*
  Lip annuli 4; body spiral … … … … … . *S. coffeae*
31. Longitudinal striae on the basal lip *<* 10 …….. 32
  Longitudinal striae on the basal lip *>* 10 … … . . 34
32. Longitudinal striae on basal lip annulus 4 … … … ………………………………. *S. imphalum*
  Longitudinal striae on basal lip annulus 6 … … . 33
33. Rectangular bend of lateral field towards ventral side of tail; stylet 24-27 *μ*m ……………… *S. sofiae* Lateral field not bent towards ventral side of tail; stylet 27-37 *μ*m………………….*S. commune*
34. Longitudinal striations on basal lip annulus 16; lip annuli 3; stylet 24-32 *μ*m……………. *S. sorghi*
  Longitudinal striae on basal lip annulus 16; lip annuli 4-5; stylet 28-32 *μ*m …………. *S. hoabinhiensis*
  Longitudinal striae on basal lip annulus *>* 20; stylet 33-38 *μ*m ……………………. *S. paludosum*
35. Basal lip annulus without longitudinal striae … . . 36
  Basal lip annulus with longitudinal striae … … . . 40
36. Scutellum *<* 2.5 *μ*m……………………..37
  Scutellum *>* 2.5 *μ*m……………………..38
37. L *<* 600 *μ*m; lip region hemispherical with irregular annulation at tail; stylet 22-24 *μ*m …… *S. insulare* L *>* 600 *μ*m; lip region subconical with regular annulation at tail; stylet 23-24 *μ*m …….. *S. impar*
38. Lip region truncate with 3 annuli . . *S. conicephalum*
  Lip region not truncate with *>*3 annuli … … … . 39
39. Lip region with 4 faint annuli; epiptygmata absent ………………………………. *S. sacchari*
  Lip region with 4-9 annuli; epiptygmata present … …………………………. *S. clathricaudatum*
40. Basal lip with *<*10 longitudinal striae … … … . . 41
  Basal lip with *>*10 longitudinal striae … … … . . 43
41. Lip annuli absent with truncate lip region … … … ………………………………. *S. truncatum*
  Lip annuli present with lip region not truncate … 42
42. Lip region not set off, with 5-7 annuli; cephalic surface divided into unequal sectors by longitudinal striae……………………… *S. anisomeristum*
  Lip region offset, with 3-4 annuli; basal lip annulus sectors with irregular number and size … … … … ……………………………. *S. cephalidum*
  Lip region slightly offset with 3-5 annuli; basal lip annulus with 6 regular sectors … … … … … … …………………………. *S. brachyurus* group
43. Scutellum *<* 4 *μ*m……………………….44
  Scutellum *>* 4 *μ*m……………………….46
44. Basal lip annulus with 10 striations; stylet 21-23 *μ*m; 3-4 lip annuli … … … … … … …*S. brevistyletum* Basal lip annulus with *>*10 striations ……….. 45
45. Stylet *<* 29 *μ*m; lip region with 3 annuli;stylet 22-25 *μ*m…………………….. *S. bangalorensis* Stylet *>* 29 *μ*m; lip region with 4 annuli … *S. unum*
46. Stylet *>* 30 *μ*m; basal lip annulus with 20-26 striations…………………… *S. magniphasma* Stylet *<* 30 *μ*m; basal lip annulus with *<*20 striations… …three very similar species: *S. ussuriensis, S. megascutatum* and *S. sanwali*
